# Global role of IGF2BP1 in controlling the expression of Wnt/β-catenin-regulated genes in colorectal cancer cells

**DOI:** 10.3389/fcell.2023.1236356

**Published:** 2023-09-27

**Authors:** Vikash Singh, Vonn Walter, Irina Elcheva, Yuka Imamura Kawasawa, Vladimir S. Spiegelman

**Affiliations:** ^1^ Division of Pediatric Hematology and Oncology, Department of Pediatrics, College of Medicine, The Pennsylvania State University, Hershey, PA, United States; ^2^ Department of Public Health Science, College of Medicine, The Pennsylvania State University, Hershey, PA, United States; ^3^ Department of Pharmacology, College of Medicine, The Pennsylvania State University, Hershey, PA, United States

**Keywords:** Wnt/β-catenin, IGF2BP1, dnTCF7L2, iCLIP, CRC

## Abstract

**Introduction:** Wnt/β-catenin signaling controls cell division and lineage specification during embryonic development, and is crucial for stem cells maintenance and gut tissue regeneration in adults. Aberrant activation of Wnt/β-catenin signaling is also essential for the pathogenesis of a variety of malignancies. The RNA-binding protein IGF2BP1 is a transcriptional target of Wnt/β-catenin signaling, normally expressed during development and often reactivated in cancer cells, where it regulates the stability of oncogenic mRNA.

**Methods:** In this study, we employed iCLIP and RNA sequencing techniques to investigate the role of IGF2BP1 in the post-transcriptional regulation of Wnt/β-catenin-induced genes at a global level within colorectal cancer (CRC) cells characterized by constitutively active Wnt/β-catenin signaling.

**Results and Discussion:** In our study, we show that, in contrast to normal cells, CRC cells exhibit a much stronger dependency on IGF2BP1 expression for Wnt/β-catenin-regulated genes. We show that both untransformed and CRC cells have their unique subsets of Wnt/β-catenin-regulated genes that IGF2BP1 directly controls through binding to their mRNA. Our iCLIP analysis revealed a significant change in the IGF2BP1-binding sites throughout the target transcriptomes and a significant change in the enrichment of 6-mer motifs associated with IGF2BP1 binding in response to Wnt/β-catenin signaling. Our study also revealed a signature of IGF2BP1-regulated genes that are significantly associated with colon cancer-free survival in humans, as well as potential targets for CRC treatment. Overall, this study highlights the complex and context-dependent regulation of Wnt/β-catenin signaling target genes by IGF2BP1 in non-transformed and CRC cells and identifies potential targets for colon cancer treatment.

## Graphical Abstract

**Figure:** Exploring the post-transcriptional regulation of three categories of Wnt/β-Catenin signaling target genes by IGF2BP1. Created with BioRender.com.

Exploring the post-transcriptional regulation of three categories of Wnt/β-Catenin signaling target genes by IGF2BP1. Created with BioRender.com.
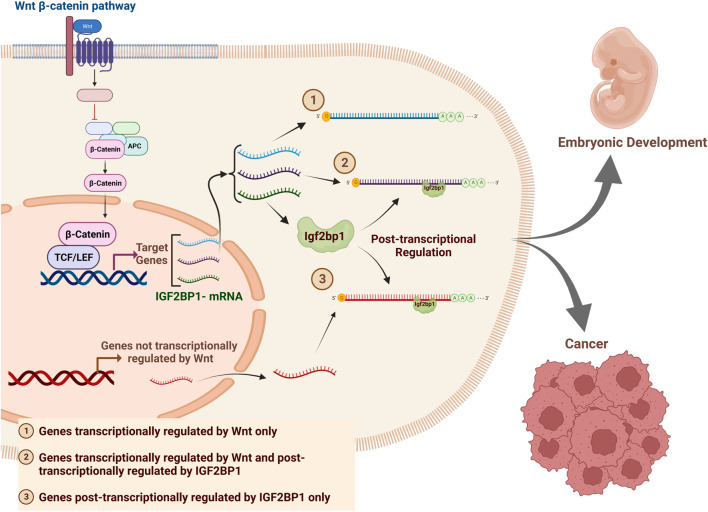

## 1 Introduction

The canonical Wnt/β-catenin signaling pathway is a highly intricate and conserved process that plays a vital role in various developmental and physiological functions, such as embryonic development, stem cell maintenance, and tissue homeostasis (Clevers and Nusse, 2012). In embryonic development, Wnt/β-catenin pathway is crucial for tissue differentiation, cell fate determination, and organogenesis. It is involved in forming the body axis, patterning tissues, and developing variety of organs including heart, brain, and limbs (Logan and Nusse, 2004). Furthermore, the Wnt pathway also plays a vital role in cancer development and progression, with dysregulation commonly observed in several malignancies. Mutations in genes associated with the Wnt pathway are often observed in different types of cancer (Niehrs, 2012). In certain cancers, such as colon cancer, mutations in APC, a negative regulator of the pathway, or in β-catenin an important transducer in the Wnt pathway, lead to constitutive activation of the Wnt pathway and the accumulation of β-catenin in the nucleus (Schneikert and Behrens, 2007). This results in the transcriptional activation of target genes involved in cell proliferation and survival, leading to uncontrolled cell growth and tumor formation. Other cancers, such as breast cancer, exhibit aberrant Wnt pathway activation through a variety of mechanisms including overexpression of Wnt ligands or receptors, mutations in Wnt pathway components, or epigenetic modifications that activate Wnt target genes. This can result in increased cell proliferation, invasion, and metastasis (MacDonald et al., 2009).

The Wnt signaling pathway is initiated when Wnt ligands bind to their receptors, which then recruit co-receptors and activate the Frizzled receptor. This initiates a cascade of events that results in the stabilization of β-catenin and its translocation into the nucleus. Once in the nucleus, β-catenin regulates target gene expression directly by interacting with the TCF/LEF family of transcription factors. The TCF/LEF proteins act as transcriptional activators or repressors, depending on their interaction with β-catenin (Behrens et al., 1996). When the Wnt signaling pathway is activated, β-catenin accumulates in the nucleus and displaces co-repressors, forming a β-catenin/TCF/LEF complex that activates target gene expression (Cadigan and Waterman, 2012). In the absence of Wnt signaling, TCF/LEF proteins bind to DNA and recruit co-repressors, such as Groucho, to inhibit gene expression. The TCF/LEF family comprises four members in mammals, each with distinct tissue-specific expression patterns and target genes, including TCF7, TCF7L1, TCF7L2, and LEF1 (Hrckulak et al., 2016).

The canonical Wnt pathway regulates the expression of several genes involved in cell proliferation and differentiation, such as c-Myc, Cyclin D1, and Axin2 (Lecarpentier et al., 2019). Moreover, the Wnt signaling pathway indirectly regulates gene expression through the regulation of oncofetal RNA-binding protein, IGF2BP1, a protein that binds to mRNA molecules and regulates their stability (Noubissi et al., 2006; Noubissi et al., 2010). For example, in response to the activation of Wnt signaling, IGF2BP1 stabilizes the mRNA of such genes as βTrCP1 and GLI1 that leads to their upregulation (Noubissi et al., 2009). Additionally, IGF2BP1 has been found to regulate the expression of other Wnt signaling components, including Wnt ligands, receptors, and downstream effectors such as c-Myc, PTGS2, GSK-3β, and β-catenin, which further increases Wnt signaling activity (Noubissi et al., 2010; Manieri et al., 2012; Fang et al., 2022). IGF2BP (Insulin-like growth factor 2 mRNA-binding protein) family of proteins comprised of a group of RNA-binding proteins that are essential for post-transcriptional gene regulation. Among these, IGF2BP1 (IMP1 or CRD-BP) has drawn a lot of interest. IGF2BP1 is distinguished by its ability to bind to specific mRNA molecules and exert control over their stability, localization, and translation (Huang et al., 2018). However, the role of IGF2BP1 in regulation of transcriptome-wide effects of Wnt/β-catenin signaling has not been elucidated.

In this study we demonstrated the crucial role of IGF2BP1 as a modulator of Wnt/β-Catenin-regulated genes on transcriptome-wide level in CRC cells. Additionally, we conducted an Individual-nucleotide resolution UV crosslinking and immunoprecipitation (iCLIP) assay to identify the direct binding targets of IGF2BP1 in wild-type MEFs and DLD1-dnTCF7L2 cells. Our study categorized the Wnt-regulated genes into three groups based on their mode of regulation (depicted in the graphical abstract). The first group comprises Wnt transcriptional target genes that are not regulated by IGF2BP1 (AXIN2, BMP4, etc.). The second group includes genes that are regulated both transcriptionally by TCF/β-catenin and post-transcriptionally by IGF2BP1, such as cMYC and MITF. The third group consists of genes that are regulated by Wnt solely post-transcriptionally by IGF2BP1 (GLI1, CD44, KRAS, PTEN, BTRC, H19) (Huang et al., 2018). This study identified new genes in all three categories of Wnt-regulated genes for non-transformed and cancer cells. We discovered 17 novel IGF2BP1 target genes regulated by Wnt/β-Catenin-IGF2BP1 axis in DLD1 cells. We used this data to analyze the expression of the 17 genes and identified five signature genes, along with IGF2BP1, that have prognostic value in colon cancer patients. A publicly available colon cancer databased was used for this analysis. This study provides novel insight into the critical role of IGF2BP1 in the Wnt/β-Catenin signaling pathway and the pathogenesis of CRC. Additionally, our findings highlight IGF2BP1 as a possible therapeutic target and a prognostic marker for colon cancer, which could be utilized to improve accuracy and even reduce cost of colon cancer detection and treatment.

## 2 Results

### 2.1 IGF2BP1 regulates transcriptomic and phenotypic attributes of β-catenin/Tcf signaling in CRC cells

**FIGURE 1:**
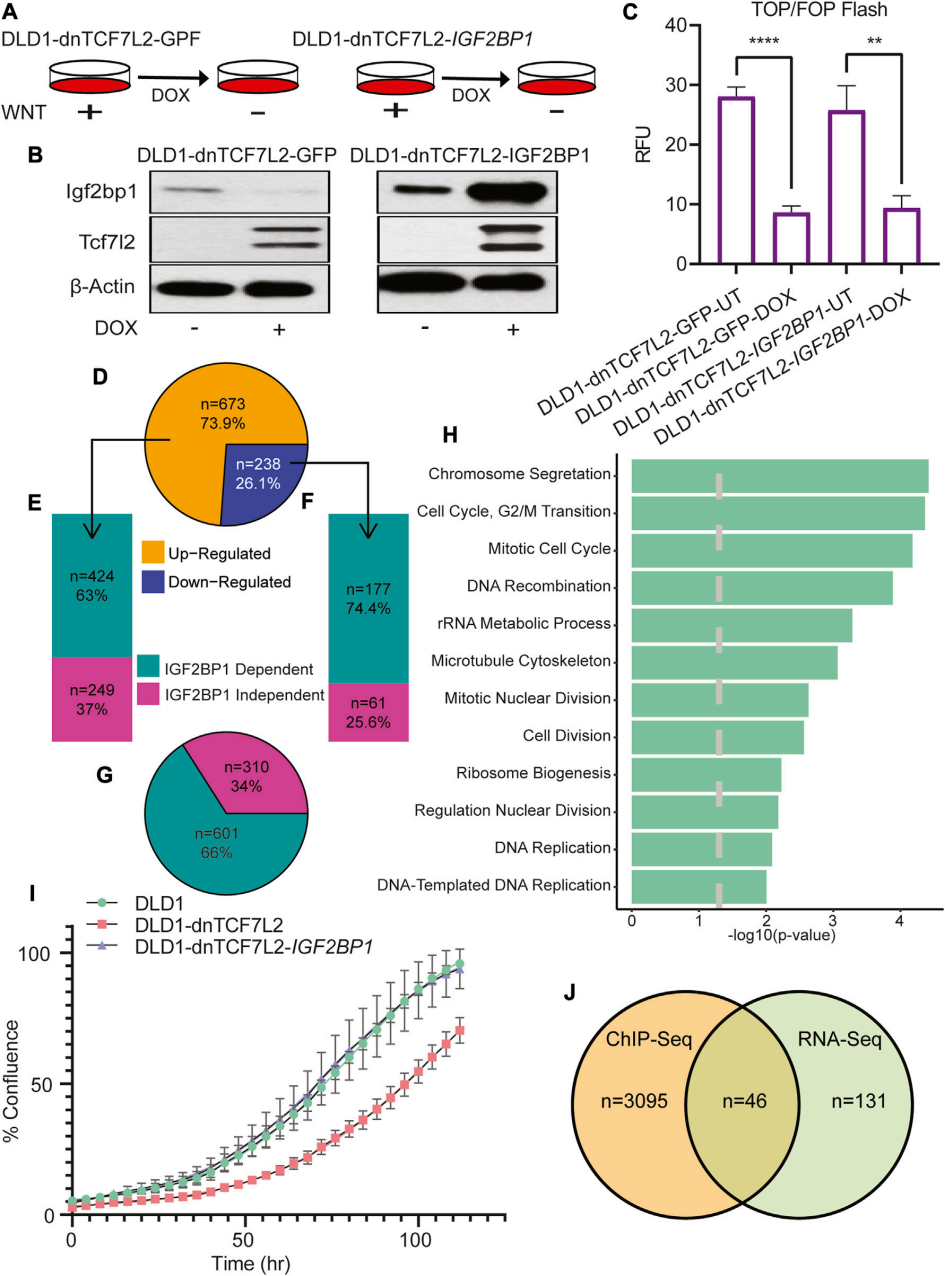
Global role of IGF2BP1 in Wnt/β-catenin-regulated genes in CRC cells. **(A)** Pictorial diagram illustrating the experimental setup: DLD1 cells were transformed and treated with doxycycline to induce the expression of dnTcf7L2 and GFP in DLD1-dnTCF7L2-GFP cells, and dnTcf7L2 and Igf2bp1 in DLD1-dnTCF7L2-IGF2BP1 cells. **(B)** Immunoblot assay showing the expression levels of Tcf7L2 and Igf2bp1 after doxycycline treatment. β-actin levels were used as a loading control. **(C)** TOP/FOP-Flash assay indicating the extent of Wnt signaling inhibition upon induction of dnTcf7L2 and GFP in DLD1-dnTCF7L2-GFP cells, and dnTcf7L2 and Igf2bp1 in DLD1-dnTCF7L2-IGF2BP1 cells. Data are presented as means ± SD (*n* = 3). **(D)** Pie chart displaying differentially expressed genes upon Wnt signaling inhibition in DLD1-dnTCF7L2-GFP cells. **(E)** Bar plot illustrating IGF2BP1-dependent/independent genes in DLD1-dnTCF7L2-IGF2BP1 cells that were upregulated in DLD1-dnTCF7L2-GFP. **(F)** Bar plot depicting IGF2BP1-dependent/independent genes in DLD1-dnTCF7L2-IGF2BP1 cells that were downregulated in DLD1-dnTCF7L2-GFP. **(G)** Pie chart showing the total number of IGF2BP1-dependent and independent genes regulated by Wnt signaling. **(H)** Gene ontology analysis of subgroup of 177 downregulated genes **(F)** regulated by Wnt signaling and controlled by IGF2BP1.**(I)** Proliferation measured as % confluence of DLD1, DLD1-dnTCF7L2, and DLD1-dnTCF7L2-IGF2BP1 using the Incucyte Live-Cell Analysis System. **(J)** Venn diagram illustrating the overlapping genes between 177 Wnt target genes **(F)** regulated by IGF2BP1 and TCF7L2-ChIP-seq genes (reported in a published article).

To investigate the role of IGF2BP1 in expression of Wnt-regulated transcriptome of CRC cells, we selected the DLD1 cell line, that harbors a homozygous APC mutation that results in constitutive activation of Wnt/β-catenin signaling (Chandra et al., 2012). We generated two distinct DLD1-derived cell lines to examine genes whose regulation by Wnt/β-Catenin signaling depends on IGF2BP1. The first cell line includes a molecular switch (dnTCF7L2) that inhibits Wnt/β-Catenin signaling temporally when activated by doxycycline (Figure 1A). The second cell line consists of doxycycline-inducible dnTCF7L2 and IGF2BP1 to achieve a simultaneous deactivation of Wnt/β-catenin dependent transcription and re-activation of IGF2BP1 expression. The inducible expression of dnTCF7L2 and IGF2BP1 in DLD1 cells was validated through immunoblot assay after doxycycline treatment (Figure 1B). We found that overexpression of cytoplasmic RNA-binding protein IGF2BP1 does not rescue the transcriptional activity of dnTCF7L2, and the disruption of β-Catenin/Tcf-dependent transcription included by dominant negative TCF7L2 is IGF2BP1-indepenent (Figure 1C), suggesting that IGF2BP1 is not involved in the ability of β-catenin/Tcf factors to bind with its DNA promoters and initiate transcription in CRC cells.

**FIGURE 2:**
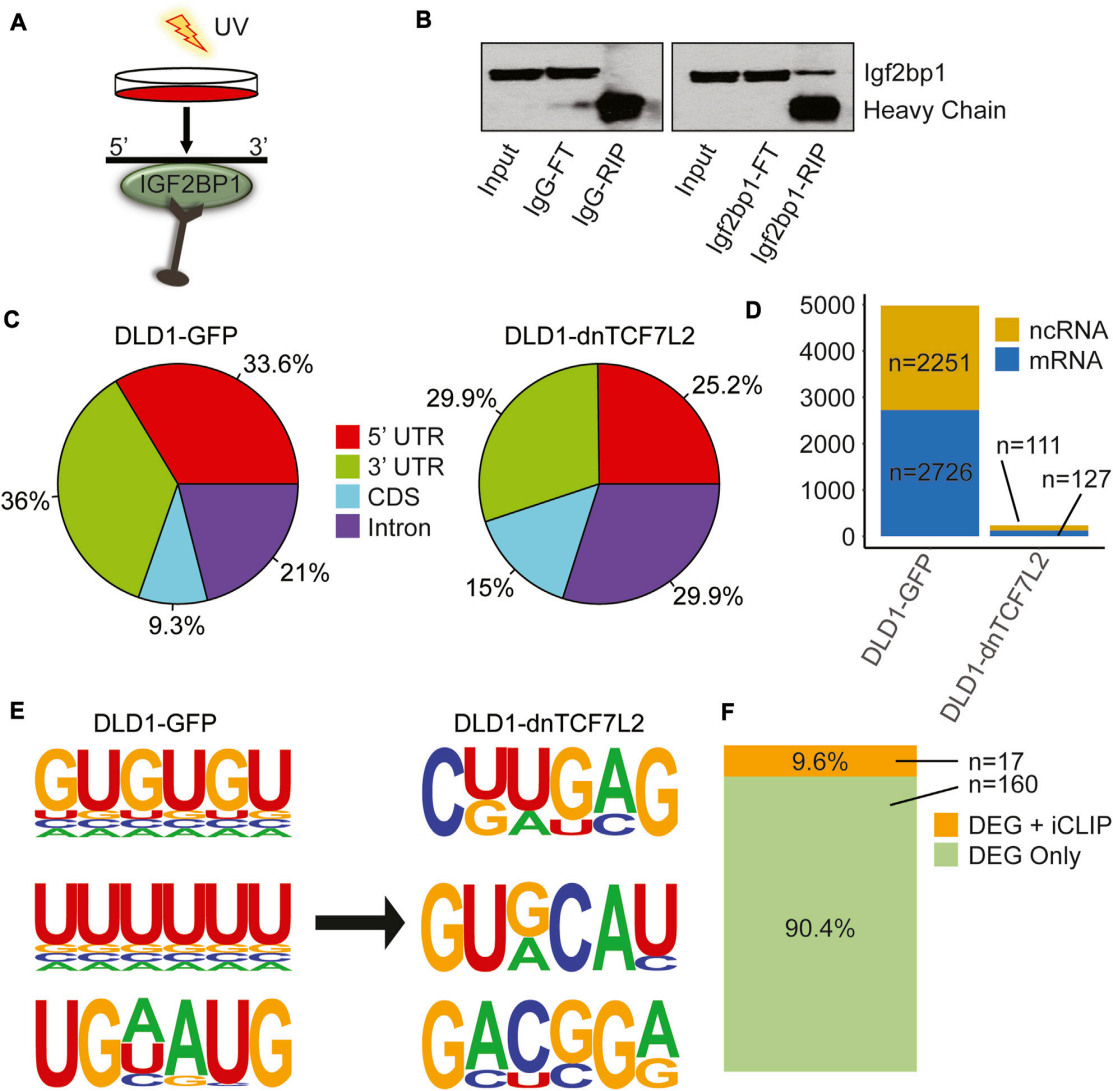
IGF2BP1 interactome and its changes in CRC cells from Wnt-active to Wnt-suppressed conditions. **(A)** iCLIP assay performed in both DLD1-GFP and DLD1-dnTCF7L2 transformed CRC cells. **(B)** Immunoblotting confirms the enrichment of Igf2bp1 in a bead-bound fraction of Crosslinked RNA-Immunoprecipitation using Igf2bp1 antibody and control IgG isotype. The iCLIP was performed with an anti-Igf2bp1 antibody or an IgG control, as indicated. Input: 2.5% of the total lysate submitted to RNA immunoprecipitation (RIP); FT (Flow-through): 5% of the extract recovered after RNA-IP; IgG-RIP/IGF2BP1-RIP: 10% of bead bound fraction. **(C)** Pie chart displaying the distribution of significantly enriched IGF2BP1 peak locations across the transcriptome in CRC cells with Wnt-active (DLD1-GFP) and Wnt-suppressed (DLD1-dnTCF7L2) conditions. **(D)** Bar graph showing the total number of significant binding peaks found in noncoding and messenger RNA in Wnt-active (DLD1-GFP) and Wnt-suppressed (DLD1-dnTCF7L2) conditions. **(E)** Three most enriched predicted IGF2BP1 motifs identified in iCLIP assays under Wnt-active (DLD1-GFP) and Wnt-suppressed (DLD1-dnTCF7L2) conditions. **(F)** Bar diagram illustrating the genes common between list of differentially expressed genes (DEG) found in RNA sequencing that are regulated by Wnt signaling + IGF2BP1 (Figure 1F), and list of genes in iCLIP analysis in Wnt-active (DLD1-GFP) cells.

Considering the role of the RNA-binding protein IGF2BP1 as a modulator of mRNA stability and translation, we conducted a comprehensive transcriptome analysis using total mRNA sequencing in CRC DLD1 cells expressing either dnTCF7L2 alone or in combination with IGF2BP1 (Figure 1D). The introduction of dnTCF7L2 to DLD1 cells, resulting in the inhibition of Wnt/β-catenin signaling, elicited alterations in the expression of 911 genes. Among these, 673 genes exhibited an increase, and 238 genes showed a decrease (Figure 1D). Intriguingly, further examination of this gene set upon overexpression of IGF2BP1 in the context of Wnt signaling blockage (DLD1-dnTCF7L2-IGF2BP1 cells) revealed that 424 out of the 673 upregulated genes (63%) (Figure 2E), as well as 177 out of the 238 downregulated genes (74.4%) (Figure 1F), changed their expression pattern differently. In aggregate, a significant fraction of the genes (66% or 601 genes) among the entire set of 911 exhibited discernibly different expression patterns after IGF2BP1 overexpression in the context of Wnt signaling inhibition (Figure 1G). A Gene Ontology (GO) analysis of the subset of genes controlled by Wnt/β-catenin and IGF2BP1 unveiled a robust enrichment of genes regulating the cell cycle (Figure 1H).

Subsequently, a proliferation assay was conducted within these cell lines, showing, the deactivation of Wnt/β-catenin signaling led to a decline in proliferation. Remarkably, upon the overexpression of IGF2BP1 into the cells with β-catenin/Tcf inhibition, the proliferation rate was reinstated to that observed in the original cell line. This underscores the potential role of IGF2BP1 in upholding the Wnt-dependent tumorigenic phenotype (Figure 1I).

To identify a subpopulation of genes that are transcriptional targets of Wnt/β-catenin signaling and post-transcriptional targets of IGF2BP1, we conducted an analysis to match the list of TCF7L2 ChIP genes from Hatzis et al.'s study with the 177 genes (IGF2BP1 dependent regulation by Wnt) obtained from our transcriptome analysis (Hatzis et al., 2008). From the cohort of 177 genes under the combined regulation of Wnt/β-catenin and IGF2BP1, we have reported 46 genes that act as direct transcriptional targets of Wnt/β-catenin. This leaves the remaining 131 genes as indirectly regulated by Wnt/β-catenin through IGF2BP1 (Figure 1J). The Gene Ontology (GO) study of the 46 genes demonstrated their major importance in the regulation of significant cellular processes, including cell motility and chemotaxis (Supplementary Table S1).

### 2.2 IGF2BP1 interactome analysis in CRC cells following dnTCF7L2-induced Wnt/β-catenin inhibition

We conducted an individual-nucleotide resolution UV crosslinking and immunoprecipitation (iCLIP) assay to investigate and compare the IGF2BP1 interactome between DLD1 cells with constitutively active Wnt/β-Catenin signaling and DLD1-dnTCF7L2 cells with inhibited Wnt/β-Catenin signaling (Figures 2A, B). We observed reduced binding for IGF2BP1 to the target mRNAs at 3′UTR and 5′UTR after inhibition of Wnt/β-catenin signaling (Figure 2C). Moreover, we observed drastic decrease in binding events for IGF2BP1 upon inhibition of Wnt/β-catenin signaling. In DLD1 cells with constitutively active Wnt signaling, we found 4,977 binding sites, comprising 2,726 on protein-coding transcripts and 2,251 on non-protein coding transcripts. Conversely, in DLD1-dnTCF7L2 cells with inhibited Wnt signaling, we identified 238 binding sites for IGF2BP1 at its interactomes, including 127 protein-coding transcripts and 111 non-protein coding transcripts. (Figure 2D). We then searched consensus binding motifs for IGF2BP1 in both conditions by analyzing the iCLIP data. In our search we found three most probable 6-mer binding motifs in both Wnt-active and Wnt-suppressed condition. Notably, we found an enrichment of 6-mer GU and UU rich motifs for Igf2bp1 binding in Wnt signaling-active DLD1 cells, whereas top 6-mer motifs in Wnt signaling-inhibited DLD1-dnTCF7L2 cells were CUUGAG and GUGCAU (Figure 2E).

Next, we compared the list of direct IGF2BP1 RNA targets (obtained from iCLIP data analysis) with the list of genes that are regulated by Wnt/β-catenin and IGF2BP1 (177 genes transcripts identified through RNA-sequencing analysis). Comparing these two lists revealed a common set of 17 genes (Figure 2F) (Supplementary Table S2). These findings suggest that IGF2BP1 regulates the expression of Wnt/β-catenin-regulated genes by both directly binding to their mRNA or indirectly through yet to be identified mechanisms.

### 2.3 Contribution of IGF2BP1 to transcriptome changes induced by Wnt ligand in non-transformed cells

**FIGURE 3:**
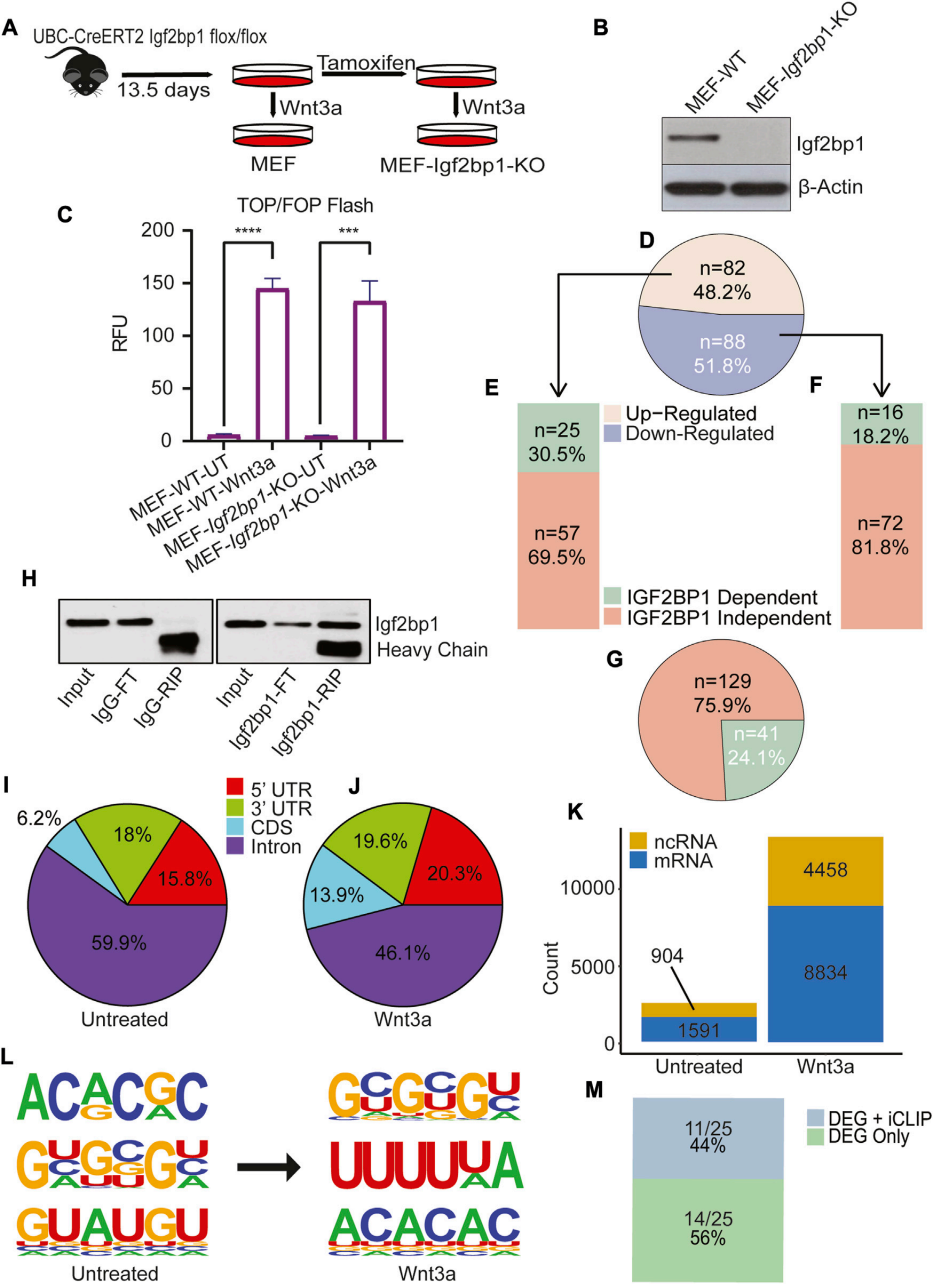
IGF2BP1 contribution to Wnt/β-catenin regulation of transcriptome in non-transformed cells. **(A)** MEF has been isolated from the 13.5 days old UBC-CreERT2 Igf2bp1 flox/flox mouse embryo, and Igf2bp1 knockout MEF cells were generated by treating cells with tamoxifen. **(B)** The Igf2bp1 knockout in MEFs was confirmed using an immunoblot assay. **(C)** The TOP/FOP Flash assay shows the extent of Wnt/β-Catenin signaling induction in MEF-wild type and MEF-Igf2bp1-knockout cells after Wnt3a ligand treatment. The data are means ± SD (*n* = 3). **(D)** Pie chart analysis of transcriptome expression showing significantly altered genes after Wnt/β-Catenin signaling induction in wild type MEF. **(E)** Bar plot showing transcriptomic expression profile of Upregulated genes shown in Figure 1D, after Wnt/β-Catenin signaling induction in Igf2bp1-knockout MEF cells. **(F)** Bar plot showing transcriptomic expression profile of downregulated genes shown in Figure 1D, after Wnt/β-Catenin signaling induction in Igf2bp1-knockout MEF. **(G)** Pie chart showing total Wnt/β-Catenin dependent transcriptomic changes dependent and independent of Igf2bp1. **(H)** Immunoblotting was used to confirm the enrichment of Igf2bp1 in a bead-bound fraction of Crosslinked RNA-Immunoprecipitation using Igf2bp1 antibody and control IgG isotype in iCLIP assaay. **(I,J)** Pie chart indicates the distribution of significantly enriched IGF2BP1 peak locations across the transcriptome in untreated **(I)**, and Wnt3a treated MEF **(J)**. **(K)** Bar graph showing the total number of significant binding peaks found on noncoding and messenger RNA in untreated and Wnt3a-treated MEF. **(L)** Three most enriched predicted Igf2bp1 motifs found in untreated and Wnt3a treated iCLIP assay analysis. **(M)** The bar diagram shows genes common between list of differentially expressed genes (DEG) found in RNA sequencing that are regulated by Wnt signaling + IGF2BP1 (Figure 3E), and list of genes found in iCLIP analysis of MEF-Wnt3a treated cells.

To investigate if our findings about the role of IGF2BP1 in regulating Wnt signaling in CRC are also applicable for non-transformed cells with the physiological Wnt/β-catenin signaling, we employed mouse embryonic fibroblasts (MEFs) with Igf2bp1^−/−^ gene knockout (Igf2bp1-KO), and a recombinant Wnt-3a ligand to study the role of Igf2bp1 upon Wnt induction *in vitro*. We have chosen MEFs for these studies for the following reasons: i) in contrast to embryonic cells, the majority of non-transformed adult cells (including intestinal cells) do not express appreciable levels of IGF2BP1; ii) CRISPR-mediated knockout of IGF2BP1 results in chronic effects on gene expression that is not suitable for these studies. Therefore, we took advantage of our tamoxifen-inducible loxP-mediated excision of Igf2bp1 that produce efficient knockout in short-term cell cultures. MEFs isolated from UBC-CreERT2:Igf2bp1^flox/flox^ mouse embryos were treated with tamoxifen to induce IGF2BP1 KO (Figures 3A, B). To activate the Wnt/β-Catenin signaling pathway, WT and IGF2BP1 KO MEFs were treated with Wnt-3a ligand. Similar to DLD1, we observed no significant difference in the extent of Wnt3a-induced β-catenin/Tcf signaling-dependent transcription activation (as measured by TOP/FOP-FLASH assay) between WT and KO IGF2BP1 MEFs (Figure 3C). These results suggest that IGF2BP1 knockout does not significantly affect transcriptional binding and activity of Wnt regulators.

To assess IGF2BP1’s role on genes activated by Wnt signals, we conducted mRNA sequencing in both wild-type (WT) and IGF2BP1 knockout (KO) MEFs. Wnt signaling was stimulated by Wnt3a treatment in both cell types. In WT MEFs, Wnt3a treatment prompted notable changes in 170 genes (with a minimum 1.5-fold difference and a false discovery rate (FDR) < 0.05). Among these, 82 genes exhibited increased expression, while 88 genes showed decreased expression (Figure 3D) compared to untreated WT MEFs. Of the upregulated genes, 30.5% (25 genes) (Figure 3E), and of the downregulated genes, 18.2% (16 genes) (Figure 3F), displayed dissimilar responses to Wnt3a treatment in IGF2BP1 KO MEFs when compared to untreated KO MEFs. Overall, 41 out of the 170 genes (24.1%) demonstrated divergent expression in IGF2BP1 KO MEFs treated with Wnt3a compared to WT MEFs treated in the same manner. This highlights that approximately one quarter of the Wnt-regulated genes are co-regulated by IGF2BP1 (Figure 3G).

The Gene ontology (GO) analysis of these 24.1% of genes regulated by both Wnt/β-Catenin and IGF2BP1 showed significant enrichment in cell processes involved in mitochondrial metabolism (Supplementary Figure S1). The XTT-Cell proliferation assay was performed at 48 and 72 h to examine the role of IGF2BP1 in MEFs. We have shown decrease in the cell proliferation rate in IGF2BP1-KO MEFs cells compared to WT MEFs cells (Supplementary Figure S5).

### 2.4 Analysis of Wnt-induced IGF2BP1 interactome in non-transformed cells

To investigate the Wnt-induced changes in RNA-binding characteristics of IGF2BP1 using MEFs Igf2bp1-KO system, we performed iCLIP analysis in MEFs treated with Wnt3a and MEFs treated with vehicle control (Figure 3H). The analysis of the overall binding preference of IGF2BP1 based on its site-specificity across RNA targets showed that IGF2BP1’s overall binding increased in the 3′UTR, 5′UTR, and CDS of its target RNA after induction with the Wnt3a ligand (Figures 3I, J).

We also found that the number of binding events for IGF2BP1 considerably increased with the activation of Wnt/β-Catenin signaling. We identified 13,292 binding sites on all identified transcriptional targets of IGF2BP1 in Wnt3a-induced MEFs, with 8,834 identified as protein-coding transcripts and 4,458 as non-protein coding transcripts, compared to 2,495 binding targets in MEFs treated with the vehicle control, with 1,591 identified as protein-coding transcripts and 904 as non-protein coding transcripts (Figure 3K). A search for sequence specificity in cross-linked regions revealed enrichment of 6-mer GC-rich and, most interestingly, polyU motifs for Igf2bp1 binding in Wnt3a-treated MEFs compared to 6-mers AC- and GU-rich motifs in vehicle-treated MEFs (Figure 3L).

To identify Wnt-regulated genes that directly depend on interactions of their mRNAs with IGF2BP1, we analyzed the intersection of IGF2BP1 target genes obtained from Wnt3a-treated MEFs iCLIP and 24.1% of genes (whose regulation by Wnt depends on IGF2BP1) obtained from transcriptome analysis. We found eleven genes in the intersection analysis, with Ptgs2 being a known binding target for IGF2BP1 (Manieri et al., 2012) (Figure 3M and Supplementary Table S3).

### 2.5 Identification of a IGF2BP1-associated prognostic gene signature for colorectal cancer

**FIGURE 4:**
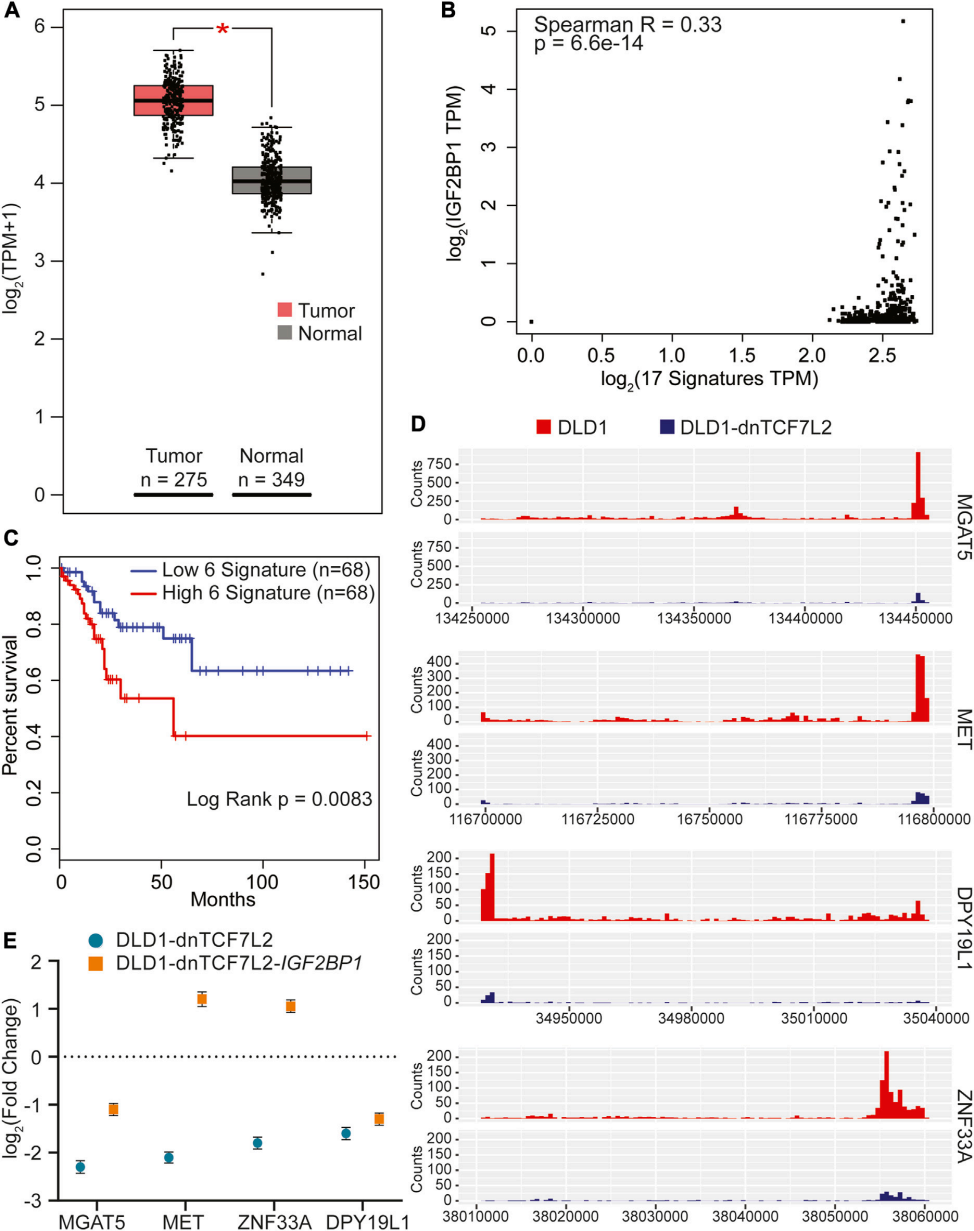
Identification of gene signature in CRC regulated by both IGF2BP1 and Wnt signaling. **(A)** The 17 overlapping genes from Figure 2F were collectively analyzed using GEPIA2 to assess their RNA expression in tumor versus normal tissue of the COAD dataset. **(B)** Spearman correlation analysis was conducted between IGF2BP1 and the 17 signature genes expressed in the COAD dataset using the GEPIA2 platform. **(C)** Kaplan-Meier curves were plotted to evaluate the disease-free survival of COAD patients expressing the 5-gene signature in conjunction with IGF2BP1, using quartile group cutoff (analysis performed using GEPIA2). **(D)** Read density tracks display the read density for IGF2BP1 in two different conditions: Wnt-active (DLD1-GFP) and Wnt-suppressed (DLD1-dnTCF7L2), spanning the 3′UTR+CDS regions of MGAT5, MET, DPY19L1, and ZNF33A. **(E)** Gene expression profiles of MGAT5, MET, DPY19L1, and ZNF33A in doxycycline-treated versus untreated conditions in DLD1-dnTCF7L2-GFP and DLD1-dnTCF7L2-IGF2BP1 cells. Data are presented as means ± SD (*n* = 3).

Taking into consideration that IGF2BP1 plays role in regulating Wnt/β-catenin-induced transcriptome in CRC cells, we, next, were seeking to identify a prognostic significance based on the 17 genes identified above as a direct IGF2BP1 targets in DLD cells. We analyzed the expression of these 17 gene’s mRNA levels in CRC patients using the Gene Expression Profiling Interactive Analysis-2 (GEPIA2) (Tang et al., 2019) online tool (Supplementary Figure S2). Our analysis revealed a significant upregulation in the combined expression of the 17 signature genes in colon adenocarcinoma tumor (COAD) TCGA cancer dataset compared to normal samples (as shown in Figure 4A). Since these genes are directly regulated by IGF2BP1, we investigated whether there is a direct correlation between these signature genes and IGF2BP1 in colon cancer patients. To achieve this, we computed a Spearman correlation in the expression of 17 gene signatures with IGF2BP1 in COAD tumor dataset using GEPIA2. We found a weak positive but significant correlation between 17 signature genes and IGF2BP1 expression (Figure 4B). To assess the prognostic value of signature gene expression, disease-free survival analysis was performed using KM plotter as a feature of GEPIA2. Out of the 17 genes that were analyzed, five genes (MGAT5, MET, ZNF33A, DPY19L1, and FAM120A), along with IGF2BP1, were found to be significantly associated with disease-free survival in COAD patient upon less expression (Figure 4C).

Next, we proceeded to verify the binding of IGF2BP1 with the aforementioned five signature genes by tracking the regions of read enrichment for IGF2BP1 binding from our DLD1 iCLIP data. Our analysis revealed that four out of five genes, namely MGAT5, MET, ZNF33A, and DPY19L1, had read enrichment for IGF2BP1 at different regions compared with the iCLIP data for DLD1-dnTCF7L2 (Figure 4D) (Supplementary Figure S4). To further validate the expression pattern of these four genes in Wnt active and Wnt suppressed conditions of CRC cells (DLD1 and DLD1-dnTCF7L2 cells), we conducted RT-qPCR analysis and found significant downregulation upon Wnt suppression. Interestingly, after overexpression of IGF2BP1 in the Wnt-suppressed background, we observed that the expression of these four genes was rescued to a varying degree (Figure 4E). To confirm whether IGF2BP1 directly binds to the mRNA of these genes, an mRNA stability test was performed in SW480-WT and SW480-IGF2BP1-KO cells. It was observed that MGAT5 and MET mRNA experienced increased degradation in SW480-IGF2BP1-KO cells compared to SW480-WT cells. This provides evidence that the binding of IGF2BP1 contributes to the stability of MGAT5 and MET mRNA (Supplementary Figure S3).

**FIGURE 5:**
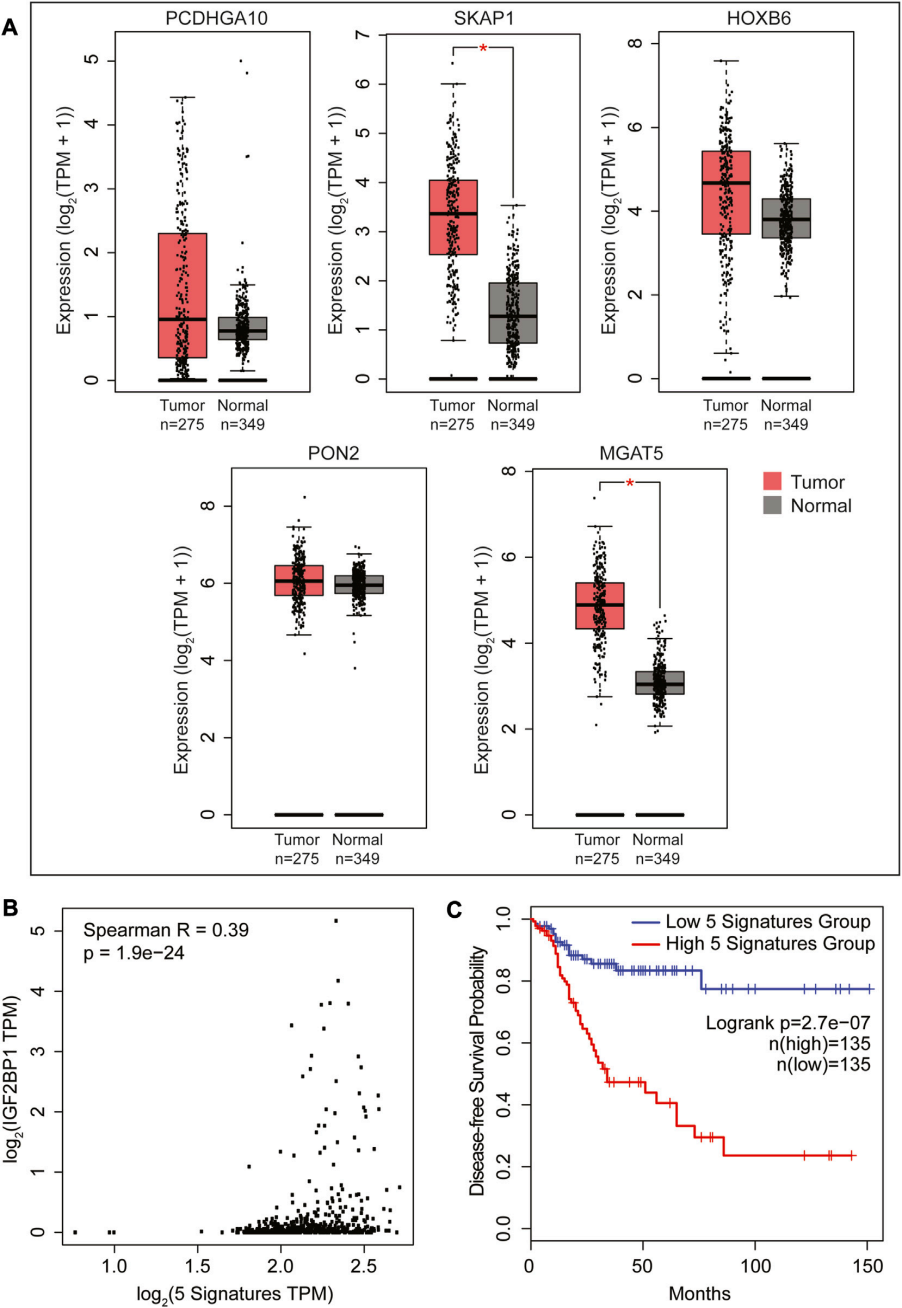
Survival analysis using the GEPIA2 platform to identify a list of the most significant survival-associated genes regulated by both IGF2BP1 and Wnt signaling in a COAD cancer dataset. **(A)** RNA expression profiles of 5 genes obtained by matching the 177 Wnt target genes (Figure 1F) regulated by IGF2BP1 with a list of the most significant survival-associated genes in the COAD cancer dataset. **(B)** Spearman correlation analysis was conducted between IGF2BP1 and the 5-gene signature expressed in the COAD dataset using the GEPIA2 platform. **(C)** Kaplan-Meier curve plotted to analyze the disease-free survival of COAD patients expressing the 5-gene signature in conjunction with IGF2BP1 using the median group cutoff (analysis performed using GEPIA2).

We utilized the online GEPIA2 tool with the COAD TCGA cancer dataset to conduct survival analysis and identify the most prominent genes associated with survival, as outlined in Supplementary Table S4. We then compared this set of 500 genes with 177 genes that were found to be regulated by IGF2BP1 and Wnt signaling in our RNA sequencing analysis on DLD1 cells. Surprisingly, we discovered six common genes, which is double than the number of genes identified when comparing the above list with genes regulated solely by Wnt signaling suggesting the importance of IGF2BP1 in Wnt-driven CRC (Supplementary Table S4). We subsequently analyzed the individual expression of each of the six genes and discovered five of them exhibited increased expression in tumor samples with MGAT5 and SKAP1 showing significant upregulation (Figure 5A). Furthermore, we investigated the correlation between these five genes and IGF2BP1 and observed a positive weak yet significant correlation (Figure 5B). To investigate the association of these five genes with poor prognosis upon higher expression, we performed disease-free survival analysis using GEPIA2. Our results showed a significant decrease in patient lifespan with higher expression of these signature genes (Figure 5C).

## 3 Discussion

Studies have shown that being regulated by Wnt/β-catenin signaling, IGF2BP1 also interacts with several components of the Wnt signaling pathway and controls their expression and function. Another potential mechanism of IGF2BP1 involvement in regulation of canonical Wnt signaling is its reported ability to regulate the expression of β-TrCP1 ubiquitin ligase receptor that target β-catenin for ubiquitination and proteasomal degradation (Noubissi et al., 2006). However, to our surprise, IGF2BP1 expression did not affect transcriptional outcome of Wnt/β-catenin signaling (assessed by TOP/FOP FLASH assay) in either non-transformed or CRC cells (Figure 1C; Figure 3C), suggesting that in our experimental settings IGF2BP1 does not regulate β-catenin/Tcf-dependent transcription.

IGF2BP1 has been shown to enhance β-TrCP1, expression in colorectal cancer, establishing its role in CRC progression (Elcheva et al., 2009; Nusse and Clevers, 2017). However, IGF2BP1 affects expression of multitude of genes and its role in Wnt-induced transcriptome on a global scale has not been elucidated. In non-transformed cells, our findings indicate that 69.5% of upregulated genes were activated by Wnt ligand treatment, independent of IGF2BP1, as illustrated in Figure 3E. AXIN2, a well-known transcriptional target of β-Catenin/Tcf7l2 regulation, was among the genes in this category (Yan et al., 2001). On the other hand, we observed that 30.5% of Wnt ligand-induced genes in non-transformed cells were regulated by IGF2BP1, with 11 of these genes being direct binding targets of IGF2BP1, as shown in our iCLIP data (Supplementary Table S3). PTGS2 is an instance of a gene that has been previously reported to be regulated by both Wnt/β-Catenin signaling and IGF2BP1 (Nunez et al., 2011; Manieri et al., 2012), underscoring the fact that some genes can be influenced by both simultaneously.

In contrast, in CRC cells, where Wnt signaling is constitutively active, we observed that only 25.6% of Wnt regulated genes were independent of IGF2BP1, as shown in Figure 1F. This gene set included AXIN2, confirming our observation of its independence of IGF2BP1 and validating the effectiveness of our model systems for studying Wnt signaling. To our surprise, the expression pattern of majority of downregulated genes (74.4%) upon β-catenin/Tcf7l2 blockage were altered by IGF2BP1 overexpression, as shown in Figure 1F. Interestingly, we have not found common genes when comparing the lists of genes regulated by Wnt signaling and IGF2BP1 in both non-transformed and CRC cells suggesting cell specific regulation of IGF2BP1. Out of the 673 genes that experienced upregulation upon suppressing Wnt/β-catenin signaling in the CRC cell line, a substantial subset (424 genes) altered their expression pattern upon introducing IGF2BP1 in the presence of Wnt blockade. Given that IGF2BP1 is not typically associated with destabilizing its target mRNA, this outcome is likely attributed to potential secondary effects. Additionally, our comparison of CRC cell RNA-seq data with published CRC cell TCF7L2 ChIP data has uncovered two categories of gene. The first involves genes transcriptionally controlled by Wnt/β-catenin (46 genes), with additional post-transcriptional regulation by IGF2BP1. The second category comprises genes indirectly influenced by Wnt/β-catenin through post-transcriptional regulation by IGF2BP1 (131 genes) (Figure 1J).

The intricate regulatory mechanism of IGF2BP1 for Wnt signaling target genes sparked our interest in investigating how IGF2BP1 interacts with its targets in diverse conditions of Wnt signaling. In order to gain deeper insights into the interaction of IGF2BP1 with its targets under different Wnt signaling conditions, we carried out iCLIP experiments on non-transformed and CRC cells. Specifically, we induced Wnt signaling in one set of experiments (non-transformed) and blocked it in another set (CRC) to study the effects on IGF2BP1-target interactions. Our findings indicate that in non-transformed cells, the interactome in Wnt-induced conditions is significantly larger than the interactome in uninduced conditions. Specifically, we observed an interactome of 13,292 in the former condition, which is more than five times greater than the 2,495 interactomes identified in the latter condition. Notably, both mRNA and ncRNA were present in these interactomes, with mRNA comprising the majority of the interactomes. Moreover, we observed an increase in both mRNA and ncRNA interactomes in response to Wnt-induction (Figure 3K). Based on our analysis of IGF2BP1 binding sites within target transcriptomes, we found that introns were the most enriched region, followed by the 3′UTR, 5′UTR, and CDS in descending order of enrichment. Notably, we also observed a significant shift in the enrichment of CDS following Wnt-induction, as shown in Figures 3I, J. Through our analysis of the target interactome, we observed that top 6-mer motifs shift from GC- and PolyU-enriched in Wnt-induced conditions compared to AC-enriched 6-mer in non-induced conditions. This suggests that the binding specificity of IGF2BP1 may be influenced by Wnt pathway. Our iCLIP analysis of colorectal cancer cells revealed that when Wnt signaling was blocked, the overall interactome of IGF2BP1 decreased significantly from 4,977 to 238 (Figure 2D). This decrease was primarily observed in the mRNA interactome. Our subsequent analysis showed that there was a major reduction in the enrichment of IGF2BP1 binding regions within the 3′UTR and 5′UTR of target mRNA (Figure 2C). Furthermore, our analysis of 6-mer motif enrichment revealed a shift from GU/UU-rich motifs to CUUGAG/GUGCAU motifs in the target interactome of IGF2BP1 under these conditions (Figure 2E). These findings suggest that Wnt signaling may play a crucial role in regulating the binding and function of IGF2BP1 in CRC cells.

Overall, in non-transformed cells, the majority of upregulated genes were activated by Wnt ligand treatment independent of IGF2BP1. However, a subset of Wnt ligand-induced genes in non-transformed cells were regulated by IGF2BP1, including 11 direct binding targets. In contrast, in CRC cells where Wnt signaling is constitutively active, a vast majority of downregulated genes upon β-catenin/Tcf7L2 blockage were found regulated by IGF2BP1, and 17 of these genes were identified as direct binding targets of IGF2BP1. Among the 177 genes affected by both Wnt signaling and IGF2BP1, only 9.6% (17 genes) were found to directly interact with IGF2BP1. This outcome could be attributed to our comparison of the CRC cell DLD1 IGF2BP1 interactome list with the known human Transcription factor list. This analysis revealed potential interactions and regulatory roles of IGF2BP1 with 139 transcription factors, which in turn might be influencing the remaining 160 genes in our transcriptome data (Supplementary Table S6). Notably, our study found that introns were the most enriched region for IGF2BP1 binding sites within target transcriptomes in both cell types. However, the enrichment pattern varied across regions, with 3′UTR and 5′UTR regions being the next most enriched regions in non-transformed cells and CDS regions being the next most enriched region in CRC cells. Additionally, our study identified a significant shift in the enrichment of 6-mer motifs associated with IGF2BP1 binding in response to Wnt signaling in both cell types, suggesting a role for Wnt signaling in regulating IGF2BP1 binding specificity. These data show that although there are significant differences in Wnt-induced IGF2BP1 interactome between two cell culture systems, there are also striking similarities, that include binding with introns and a shift between GUGU and UUUU motifs upon WNT activation in both systems. Our study also revealed that the overall interactome of IGF2BP1 decreased significantly in response to Wnt-blockade in CRC cells, with a major reduction in the enrichment of IGF2BP1 binding regions within the 3′UTR and 5′UTR of target mRNA. The lower frequency of binding events with induction of dnTCF7L2 is concomitant with reduced IGF2BP1 levels, underscoring the functional importance of IGF2BP1’s modulation by the Wnt signaling pathway. The binding pattern can also be contingent upon IGF2BP1 levels, capturing distinct subpopulations of binding entities. Our study elucidated functional distinctions driven by the dynamic alterations in IGF2BP1 levels, exemplifying the changes induced by dnTCF7L2. This explanation holds true for non-transformed cells as well. Overall, the study highlights the complex and context-dependent regulation of Wnt signaling target genes by IGF2BP1 in non-transformed cells, with the emphasis on metabolic gene set, and in CRC cells, with the emphasis on maintaining proliferation and cell division (assessed with GO KEGG analysis).

Further, matching the IGF2BP1 interactome data with transcriptomic data, we have found 17 common genes, including MGAT5 and MET, which showed a higher degree of expression in colon tumors analyzed from the TCGA database. Our study uncovered a five-gene signature, including IGF2BP1, which showed a significant association with disease-free survival in human CRCs. MGAT5 was found to be a direct target of IGF2BP1 (Yang et al., 2021), and increased MGAT5 expression in CRC cells has been associated with enhanced N-glycosylation of key proteins and poor prognosis in CRC patients (de Freitas Junior and Morgado-Diaz, 2016). In this study we identified MET as a new target of IGF2BP1. MET is known to play a critical role in mediating cross-talk between the MET receptor and Wnt signaling and represents a potential therapeutic target for cancer treatment (Boon et al., 2002). As IGF2BP1 has an oncofetal expression pattern and is overexpressed in several types of cancers, including CRC (Ioannidis et al., 2003). So, we have conducted a distinct analysis to compare the most significant genes associated with survival obtained from the human colon cancer patient dataset with our transcriptomic data. In this analysis, we identified five additional genes that are linked to poor prognosis in colon cancer patients, as illustrated in Figure 5C. It is noteworthy that MGAT5 was also present in this analysis, providing further evidence of its prominent role in Wnt-IGF2BP1 driven CRC.

Overall, our study emphasizes the importance of IGF2BP1 in regulating Wnt target genes. Activation of the Wnt/β-Catenin-IGF2BP1 pathway in cancer cells leads to increased gene expression of a subset of genes, promoting an aggressive tumor phenotype and unfavorable prognostic outcomes.

## 4 Materials and methods

### 4.1 Cell culture and reagents

DLD1 cells, a human colon cancer cell line, and its transformed counterparts were cultured and maintained as monolayers in RPMI medium (VWR International) supplemented with 10% (v/v) fetal bovine serum (Gibco, Life Technologies, Inc.) and 100 units/ml penicillin and streptomycin (Corning). The cells were incubated at 37°C with 5% CO2. *Mycoplasma* contamination was assessed in all cell lines using the MycoAlert PLUS *mycoplasma* detection kit (Lonza).

### 4.2 Preparation of MEF cell cultures

All animal studies were conducted following approved protocols by the Institutional Animal Care and Usage Committee of Penn State College of Medicine, Hershey. Embryos from UBC-CreERT2:Igf2bp1flox/flox transgenic mice were isolated between E13.5. After removing the heads, tails, limbs, and most internal organs, the embryos were minced and trypsinized for 20 min, then seeded into 100 mm cell culture dishes coated with gelatin in 10 mL of complete MEF media (DMEM supplemented with 10% heat-inactivated FBS). When the cells reached confluency, they were split at 1:2–1:3 ratios. This process was repeated two or three times to obtain a morphologically homogeneous culture, after which the cells were either frozen or expanded for further studies. Tamoxifen treatment was used to induce Igf2bp1 knockout in MEF cells. To activate Wnt signaling in MEF cells, Wnt3a ligand was employed.

### 4.3 Generation of dnTCF7L2 and IGF2BP1 overexpression cell lines

A doxycycline-inducible overexpression lentiviral plasmid for dnTCF7L2 was constructed by cloning PCR-amplified TCF4-DN, from pLX303 plasmid (a gift from William Hahn, Addgene plasmid #42592; http://n2t.net/addgene:42592; RRID: Addgene_42592), into the pInducer-20 vector. A doxycycline-inducible overexpression lentiviral plasmid for IGF2BP1 was created by cloning PCR-amplified human IGF2BP1 ORF into the pInducer-21 vector. The pInducer20 and pInducer21 were gifted from Stephen Elledge and Thomas Westbrook (Addgene plasmid # 44012; http://n2t.net/addgene:44012; RRID: Addgene_44012), (Addgene plasmid # 46948; http://n2t.net/addgene:46948; RRID: Addgene_46948) (Meerbrey et al., 2011). Lentivirus particles were produced from the above plasmids in Lenti-X 293T cells and used to transduce DLD1 CRC cells, resulting in stable DLD1-dnTCF7L2-GFP and DLD1-dnTCF7L2-IGF2BP1 Dox-inducible cell lines.

### 4.4 SDS-PAGE and immunoblotting analysis

For SDS-PAGE and immunoblotting, DLD1-dnTCF7L2-GFP, DLD1-dnTCF7L2-IGF2BP1, MEF wild-type, and MEF-Igf2bp1-KO cells were harvested, and whole-cell extract (WCE) was prepared by adding 1 mL of cell lysis buffer per 5 × 107 cells. WCE protein content was quantified using a Bio-Rad protein estimation kit. The WCE was subjected to SDS-PAGE (10%) and subsequently transferred to a membrane for immunoblotting. Blots were probed with antibodies against Igf2bp1, Myc, and β-actin obtained from Cell Signaling. Horseradish peroxidase-conjugated secondary antibodies (anti-mouse or anti-rabbit) were used to detect the respective primary antibodies. Quantitative luminescence of immunoblots was performed using ImageJ software.

### 4.5 Real-time PCR

Quantitative real-time PCR was performed to measure mRNA levels. Total RNA was extracted using a RNeasy minikit from Qiagen, and cDNA was synthesized using an iScript cDNA synthesis kit (Bio-Rad) following the manufacturer’s instructions. Real-time PCR was conducted using a CFX-96 RT-PCR machine with Bio-Rad SYBR Green mix. The PCR conditions included an initial denaturation at 95°C for 3 min, followed by 40 amplification cycles consisting of denaturation at 95°C for 20 s, annealing at 58°C for 20 s, and elongation at 72°C for 20 s. Analysis of the results was performed using CFX software from Bio-Rad to determine the fold change in expression of specific genes. Three independent experiments were carried out, with each sample run in triplicate.

### 4.6 TOP/FOP luciferase reporter assay

To evaluate the transcriptional activity of Wnt/β-catenin in MEF-WT, MEF-Igf2bp1-KO, DLD1-dnTCF7L2-GFP, and DLD1-dnTCF7L2-IGF2BP1 cells, we employed the TOP/FOP reporter system in conjunction with the dual-luciferase kit (Dual-GloTM Luciferase Assay System, Promega, Madison, WI, USA). All cells (10 × 106/500 μL MEF medium/RPMI) were transiently transfected with 1 μg of a constitutively active vector encoding Renilla luciferase (Promega) and 10 μg of a β-catenin-responsive firefly luciferase reporter plasmid (TopFlash) or the negative control (FopFlash) using the MEF transfection reagent (Altogen Biosystems) or lipofectamine 2000 (Invitrogen). After 24 h, the cells were harvested, and both firefly and Renilla luciferase activities were measured in duplicate or triplicate according to the manufacturer’s instructions. Firefly luciferase activity was normalized against Renilla luciferase activity, and the fold increase in TOPFlash activity compared to FOPFlash was determined.

### 4.7 Individual-nucleotide resolution UV crosslinking and immunoprecipitation (iCLIP)

Briefly, MEF-UT/MEF-Wnt3a/DLD1-GFP/DLD1-dnTCF7L2 cells were exposed to UV-C light to induce covalent cross-linking between proteins and nucleic acids *in vivo*. After cell lysis, RNA was partially fragmented using micrococcal nuclease, and IGF2BP1-RNA complexes were immunopurified using anti-IGF2BP1 antibody (MBL International Corporation) immobilized on protein A-coated Dynabeads (Invitrogen). Following stringent washing and dephosphorylation (FastAP, Fermentas), RNAs were ligated at their 3′ ends to a 3′ preadenylated RNA adaptor, radioactively labeled, separated by MOPS-based protein gel electrophoresis, and transferred to a nitrocellulose membrane. Protein-RNA complexes located 15–80 kDa above free protein were excised from the membrane, and RNA was recovered through proteinase K digestion under denaturing conditions (3.5 M urea). Reverse transcription was performed using oligonucleotides containing two inversely oriented adaptor regions, separated by a BamHI restriction site and a barcode region at their 5′ end. The barcode region contained a 4-nt experiment-specific barcode within a 5-nt random barcode to identify individual cDNA molecules. The cDNA molecules were size purified using denaturing PAGE, circularized using CircLigase II (Epicenter, Illumina), annealed to an oligonucleotide complementary to the restriction site, and cleaved using BamHI (New England Biolabs Inc.). The linearized cDNAs were then PCR-amplified using AccuPrime Super Mix I (Invitrogen) with primers (IDT) complementary to the adaptor regions and subjected to high-throughput sequencing using the Illumina HiSeq platform. For a more detailed description of the iCLIP protocol, refer to the published literature (Huppertz et al., 2014). Supplementary Table S5 contains detailed list of reagents used in the iCLIP and other experiment.

### 4.8 The iCLIP and RNA sequencing data analysis

All of the analyses described below were performed separately for human and mouse samples using the pipeline detailed in (Busch et al., 2020). Here we provide a brief overview. Initial quality checks were performed on the multiplexed fastq files using FastQC 0.11.8 (Krueger and Andrews, 2011). Next, the FASTX-Toolkit (Hanoon, 2010) was implemented to trim reads of the barcode region of length 9, followed by restricting to barcode regions that have at least a minimum Phred score of 10 at all positions. The resulting read IDs were written to a temporary file. Read IDs containing whitespaces or special characters were removed, then seqtk 1.3 was used to create a new fastq file containing the corresponding reads. Demultiplexing and adapter trimming were performed with Flexbar 3.5.0 (Roehr et al., 2017). No mismatches for barcode matching were allowed, and only 1 nucleotide of overlap between the read 3’ end and the beginning of the adapter was required. Trimmed reads with length at least 15 were retained for further analyses. This created separate fastq files for each experimental condition and replicate.

The STAR 2.7.3a aligner (Dobin et al., 2013) was used to make index files based on primary fasta reference files (GRCh38. primary_assembly.genome.fa for human, GRCm38. primary_assembly.genome.fa for mouse) and gene annotation files (gencode.v35. annotation.gtf for human, gencode.vM25. annotation.gtf for mouse). The alignment was then performed with STAR to produce bam files based on the parameters in Busch et al. and a maximum read length of 52. UMI-tools 1.0.0 (Smith et al., 2017) was applied to remove technical duplicate reads from the bam files that arise during PCR amplification. Duplicate reads were identified based on their read ID using the --method unique option.

Next, the duplicate-removed bam files were converted to bed format using Bedtools 2.29.2 (Quinlan and Hall, 2010). These bed files were shifted by 1 nucleotide in the 5’ direction in order to correctly identify crosslinked nucleotides. The shifted bed files were used to create bedgraph coverage files, a process that was performed separately for each strand. Bigwig format coverage files were also created.

Using PureCLIP 1.3.1 (Krakau et al., 2017), the bam files were processed to make bed files containing sites that were associated with a significant crosslinked signal. Post-processing steps were applied to these bed files with the goal of resizing the binding sites to have uniform width 9 nucleotides, as was done in the Busch et al. manuscript. These bed files were the basis of numerous downstream analyses that we describe below.

The RCAS R package (Uyar et al., 2017) was used to determine whether binding sites were found in the 5′ UTR, 3′ UTR, coding sequence, or intron of mapped genes. Gene ontology analyses based on genes with 3′ UTR binding was performed with the topGO R package. Coverage plots for select genes of interest were created with the ggcoverage R package using the bigwig files described above. The genomic intervals corresponding to the 3’ UTR + coding regions used in the coverage plots were obtained from the UCSC Table Browser (Karolchik et al., 2004). Supplementary coverage plots that include information about gene structure were created using the GenVis R package (Skidmore et al., 2016) coverage files produced by bedtools, and genomic intervals for canonical transcripts obtained from the Ensembl website. HOMER software (Heinz et al., 2010) was applied to perform RNA-based motif analyses.

After filtering lowly expressed genes, the limma R package together with the voom transformation (Law et al., 2014) was used to perform differential expression analyses for comparisons of interest using files containing gene-level read counts from RNA sequencing. Differentially expressed genes were identified using a false discovery rate threshold of q = 0.05 and a log_2_-fold change of $\pm \log_{2}\left(1.5\right)$ . R 4.0.5 and R 4.2.0 (Team, 2022) were used to perform the analyses described above, process data sets, and create figures.

### 4.9 Expression and survival analysis of Wnt regulated and IGF2BP1 controlled genes in colon adenocarcinoma

We utilized the online resource Gene Expression Profiling and Interactive Analyses (GEPIA) developed (Tang et al., 2017) for gene expression analysis. An updated version of this tool, called GEPIA2, was utilized in our study (Tang et al., 2019). GEPIA2 incorporated a dataset comprising 275 COAD cases from the TCGA database and 349 normal colon samples from the TCGA and Genome Tissues Expression (GETx) database (Consortium, 2015). To investigate the differential expression of Wnt-regulated and IGF2BP1-controlled genes, we performed gene expression analysis using GEPIA2. The expression levels were presented using boxplots, and the values were transformed into log2 of transcript count per million [log2 (TPM + 1)]. These boxplots allowed us to visualize the expression levels of either all genes or a specific gene signature group. Furthermore, we conducted survival analyses, including disease-free survival (DFS), using the GEPIA2 database. Patients were categorized into two groups (high and low expression) based on the quartile or median expression level of COAD in cancer samples. The Kaplan-Meier methods were employed to generate survival curves, and we calculated the associated log rank test *p*-values. Statistical significance was determined using a threshold of *p* < 0.05, indicating significant differences in expression and survival outcomes.

## Data Availability

The data presented in the study are deposited in the Gene Expression Omnibus (GEO) repository, accession number is GSE237666.

## References

[B1] Behrens J. Von Kries J. P. Kuhl M. Bruhn L. Wedlich D. Grosschedl R. (1996). Functional interaction of beta-catenin with the transcription factor LEF-1. Nature 382, 638–642. 10.1038/382638a0 8757136

[B2] Boon E. M. Van Der Neut R. Van De Wetering M. Clevers H. Pals S. T. (2002). Wnt signaling regulates expression of the receptor tyrosine kinase met in colorectal cancer. Cancer Res. 62, 5126–5128. 12234972

[B3] Busch A. Bruggemann M. Ebersberger S. Zarnack K. (2020). iCLIP data analysis: A complete pipeline from sequencing reads to RBP binding sites. Methods 178, 49–62. 10.1016/j.ymeth.2019.11.008 31751605

[B4] Cadigan K. M. Waterman M. L. (2012). TCF/LEFs and Wnt signaling in the nucleus. Cold Spring Harb. Perspect. Biol. 4, a007906. 10.1101/cshperspect.a007906 23024173 PMC3536346

[B5] Chandra S. H. Wacker I. Appelt U. K. Behrens J. Schneikert J. (2012). A common role for various human truncated adenomatous polyposis coli isoforms in the control of beta-catenin activity and cell proliferation. PLoS One 7, e34479. 10.1371/journal.pone.0034479 22509309 PMC3317983

[B6] Clevers H. Nusse R. (2012). Wnt/β-catenin signaling and disease. Cell 149, 1192–1205. 10.1016/j.cell.2012.05.012 22682243

[B7] Consortium G. T. (2015). Human genomics. the genotype-tissue expression (GTEx) pilot analysis: multitissue gene regulation in humans. Science 348, 648–660. 10.1126/science.1262110 25954001 PMC4547484

[B8] De Freitas Junior J. C. Morgado-Diaz J. A. (2016). The role of N-glycans in colorectal cancer progression: potential biomarkers and therapeutic applications. Oncotarget 7, 19395–19413. 10.18632/oncotarget.6283 26539643 PMC4991391

[B9] Dobin A. Davis C. A. Schlesinger F. Drenkow J. Zaleski C. Jha S. (2013). Star: ultrafast universal RNA-seq aligner. Bioinformatics 29, 15–21. 10.1093/bioinformatics/bts635 23104886 PMC3530905

[B10] Elcheva I. Goswami S. Noubissi F. K. Spiegelman V. S. (2009). CRD-BP protects the coding region of betaTrCP1 mRNA from miR-183-mediated degradation. Mol. Cell 35, 240–246. 10.1016/j.molcel.2009.06.007 19647520 PMC2742352

[B11] Fang F. Guo C. Zheng W. Wang Q. Zhou L. (2022). Exosome-mediated transfer of miR-1323 from cancer-associated fibroblasts confers radioresistance of C33A cells by targeting PABPN1 and activating wnt/β-catenin signaling pathway in cervical cancer. Reprod. Sci. 29, 1809–1821. 10.1007/s43032-021-00820-y 35334101

[B12] Hanoon G. J. (2010). FASTX-Toolkit. Available at: http://hannonlab.cshl.edu/fastx_toolkit .

[B13] Hatzis P. Van Der Flier L. G. Van Driel M. A. Guryev V. Nielsen F. Denissov S. (2008). Genome-wide pattern of TCF7L2/TCF4 chromatin occupancy in colorectal cancer cells. Mol. Cell Biol. 28, 2732–2744. 10.1128/MCB.02175-07 18268006 PMC2293123

[B14] Heinz S. Benner C. Spann N. Bertolino E. Lin Y. C. Laslo P. (2010). Simple combinations of lineage-determining transcription factors prime cis-regulatory elements required for macrophage and B cell identities. Mol. Cell 38, 576–589. 10.1016/j.molcel.2010.05.004 20513432 PMC2898526

[B15] Hrckulak D. Kolar M. Strnad H. Korinek V. (2016). TCF/LEF transcription factors: An update from the internet resources, Basel: Cancers. 10.3390/cancers8070070 PMC4963812 27447672

[B16] Huang X. Zhang H. Guo X. Zhu Z. Cai H. Kong X. (2018). Insulin-like growth factor 2 mRNA-binding protein 1 (IGF2BP1) in cancer. J. Hematol. Oncol. 11, 88. 10.1186/s13045-018-0628-y 29954406 PMC6025799

[B17] Huppertz I. Attig J. D'Ambrogio A. Easton L. E. Sibley C. R. Sugimoto Y. (2014). iCLIP: protein-RNA interactions at nucleotide resolution. Methods 65, 274–287. 10.1016/j.ymeth.2013.10.011 24184352 PMC3988997

[B18] Ioannidis P. Mahaira L. Papadopoulou A. Teixeira M. R. Heim S. Andersen J. A. (2003). CRD-BP: A c-myc mRNA stabilizing protein with an oncofetal pattern of expression. Anticancer Res. 23, 2179–2183. 12894594

[B19] Karolchik D. Hinrichs A. S. Furey T. S. Roskin K. M. Sugnet C. W. Haussler D. (2004). The UCSC Table Browser data retrieval tool. Nucleic Acids Res. 32, D493–D496. 10.1093/nar/gkh103 14681465 PMC308837

[B20] Krakau S. Richard H. Marsico A. (2017). PureCLIP: capturing target-specific protein-RNA interaction footprints from single-nucleotide CLIP-seq data. Genome Biol. 18, 240. 10.1186/s13059-017-1364-2 29284540 PMC5746957

[B21] Krueger F. Andrews S. R. (2011). Bismark: A flexible aligner and methylation caller for bisulfite-seq applications. Bioinformatics 27, 1571–1572. 10.1093/bioinformatics/btr167 21493656 PMC3102221

[B22] Law C. W. Chen Y. Shi W. Smyth G. K. (2014). voom: precision weights unlock linear model analysis tools for RNA-seq read counts. Genome Biol. 15, R29. 10.1186/gb-2014-15-2-r29 24485249 PMC4053721

[B23] Lecarpentier Y. Schussler O. Hebert J. L. Vallee A. (2019). Multiple targets of the canonical WNT/β-Catenin signaling in cancers. Front. Oncol. 9, 1248. 10.3389/fonc.2019.01248 31803621 PMC6876670

[B24] Logan C. Y. Nusse R. (2004). The Wnt signaling pathway in development and disease. Annu. Rev. Cell Dev. Biol. 20, 781–810. 10.1146/annurev.cellbio.20.010403.113126 15473860

[B25] Macdonald B. T. Tamai K. He X. (2009). Wnt/beta-catenin signaling: components, mechanisms, and diseases. Dev. Cell 17, 9–26. 10.1016/j.devcel.2009.06.016 19619488 PMC2861485

[B26] Manieri N. A. Drylewicz M. R. Miyoshi H. Stappenbeck T. S. (2012). Igf2bp1 is required for full induction of Ptgs2 mRNA in colonic mesenchymal stem cells in mice. Gastroenterology 143, 110–121. 10.1053/j.gastro.2012.03.037 22465430 PMC3383944

[B27] Meerbrey K. L. Hu G. Kessler J. D. Roarty K. Li M. Z. Fang J. E. (2011). The pINDUCER lentiviral toolkit for inducible RNA interference *in vitro* and *in vivo* . Proc. Natl. Acad. Sci. U. S. A. 108, 3665–3670. 10.1073/pnas.1019736108 21307310 PMC3048138

[B28] Niehrs C. (2012). The complex world of WNT receptor signalling. Nat. Rev. Mol. Cell Biol. 13, 767–779. 10.1038/nrm3470 23151663

[B29] Noubissi F. K. Elcheva I. Bhatia N. Shakoori A. Ougolkov A. Liu J. (2006). CRD-BP mediates stabilization of betaTrCP1 and c-myc mRNA in response to beta-catenin signalling. Nature 441, 898–901. 10.1038/nature04839 16778892

[B30] Noubissi F. K. Goswami S. Sanek N. A. Kawakami K. Minamoto T. Moser A. (2009). Wnt signaling stimulates transcriptional outcome of the Hedgehog pathway by stabilizing GLI1 mRNA. Cancer Res. 69, 8572–8578. 10.1158/0008-5472.CAN-09-1500 19887615 PMC2783483

[B31] Noubissi F. K. Nikiforov M. A. Colburn N. Spiegelman V. S. (2010). Transcriptional regulation of CRD-BP by c-myc: implications for c-myc functions. Genes Cancer 1, 1074–1082. 10.1177/1947601910395581 21779431 PMC3092266

[B32] Nunez F. Bravo S. Cruzat F. Montecino M. De Ferrari G. V. (2011). Wnt/β-catenin signaling enhances cyclooxygenase-2 (COX2) transcriptional activity in gastric cancer cells. PLoS One 6, e18562. 10.1371/journal.pone.0018562 21494638 PMC3071840

[B33] Nusse R. Clevers H. (2017). Wnt/β-Catenin signaling, disease, and emerging therapeutic modalities. Cell 169, 985–999. 10.1016/j.cell.2017.05.016 28575679

[B34] Quinlan A. R. Hall I. M. (2010). BEDTools: A flexible suite of utilities for comparing genomic features. Bioinformatics 26, 841–842. 10.1093/bioinformatics/btq033 20110278 PMC2832824

[B35] Roehr J. T. Dieterich C. Reinert K. (2017). Flexbar 3.0 - SIMD and multicore parallelization. Bioinformatics 33, 2941–2942. 10.1093/bioinformatics/btx330 28541403

[B36] Schneikert J. Behrens J. (2007). The canonical Wnt signalling pathway and its APC partner in colon cancer development. Gut 56, 417–425. 10.1136/gut.2006.093310 16840506 PMC1856802

[B37] Skidmore Z. L. Wagner A. H. Lesurf R. Campbell K. M. Kunisaki J. Griffith O. L. (2016). GenVisR: genomic visualizations in R. Bioinformatics 32, 3012–3014. 10.1093/bioinformatics/btw325 27288499 PMC5039916

[B38] Smith T. Heger A. Sudbery I. (2017). UMI-Tools: modeling sequencing errors in unique molecular identifiers to improve quantification accuracy. Genome Res. 27, 491–499. 10.1101/gr.209601.116 28100584 PMC5340976

[B39] Tang Z. Kang B. Li C. Chen T. Zhang Z. (2019). GEPIA2: an enhanced web server for large-scale expression profiling and interactive analysis. Nucleic Acids Res. 47, W556-W560–W560. 10.1093/nar/gkz430 31114875 PMC6602440

[B40] Tang Z. Li C. Kang B. Gao G. Li C. Zhang Z. (2017). Gepia: A web server for cancer and normal gene expression profiling and interactive analyses. Nucleic Acids Res. 45, W98-W102–W102. 10.1093/nar/gkx247 28407145 PMC5570223

[B41] Team R. C. (2022). R: A language and environment for statistical computing. Vienna, Austria: R Foundation for Statistical Computing.

[B42] Uyar B. Yusuf D. Wurmus R. Rajewsky N. Ohler U. Akalin A. (2017). Rcas: an RNA centric annotation system for transcriptome-wide regions of interest. Nucleic Acids Res. 45, e91. 10.1093/nar/gkx120 28334930 PMC5449606

[B43] Yan D. Wiesmann M. Rohan M. Chan V. Jefferson A. B. Guo L. (2001). Elevated expression of axin2 and hnkd mRNA provides evidence that Wnt/beta -catenin signaling is activated in human colon tumors. Proc. Natl. Acad. Sci. U. S. A. 98, 14973–14978. 10.1073/pnas.261574498 11752446 PMC64968

[B44] Yang Y. Wu J. Liu F. He J. Wu F. Chen J. (2021). IGF2BP1 promotes the liver cancer stem cell phenotype by regulating MGAT5 mRNA stability by m6A RNA methylation. Stem Cells Dev. 30, 1115–1125. 10.1089/scd.2021.0153 34514861

