# Supplementary material for: Global role of IGF2BP1 in controlling the expression of Wnt/β-catenin-regulated genes in colorectal cancer cells
Source: Front Cell Dev Biol. 2023 Sep 27;11:1236356. doi: 10.3389/fcell.2023.1236356 (PMC10565211; doi:10.3389/fcell.2023.1236356)
Supplement: Supplementary file 8 [file DataSheet1.docx]

Supplementary Material

Global role of IGF2BP1 in controlling the expression of Wnt/β-catenin-regulated genes

Vikash Singh^1^, Vonn Walter^2^, Irina Elcheva^1^, Yuka Imamura Kawasawa^3^, Vladimir S. Spiegelman^1*^

*** Correspondence: Vladimir S. Spiegelman** - Division of Pediatric Hematology and Oncology, Department of Pediatrics, The Pennsylvania State University College of Medicine Hershey, PA, USA; E-mail: [vspiegelman@pennstatehealth.psu.edu](mailto:vspiegelman@pennstatehealth.psu.edu); Phone: 1-717-531-6719.

## Supplementary Figures


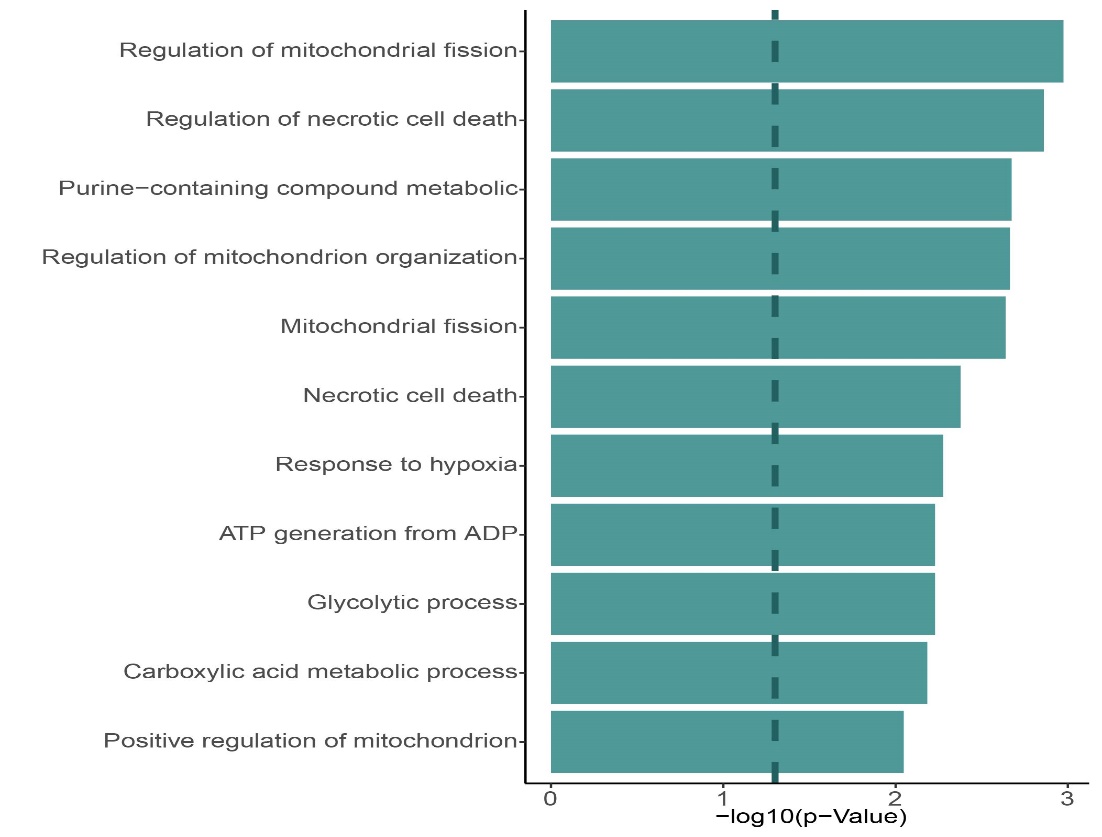


**Supplementary Figure 1.** Gene ontology analysis of subgroup of genes regulated by Wnt signaling and controlled by IGF2BP1 in non-transformed MEF.


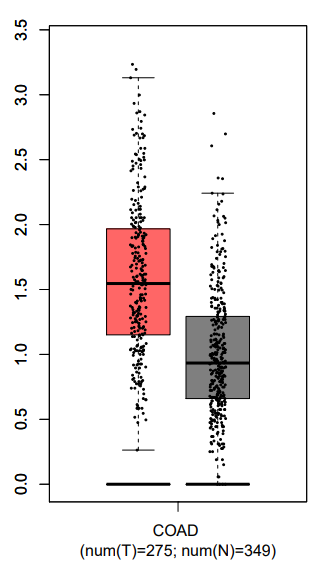


**CCDC150**


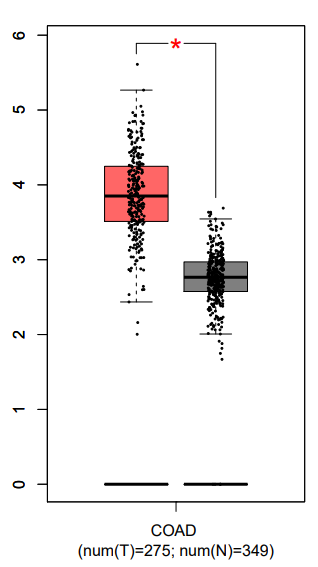


**DPY19L1**


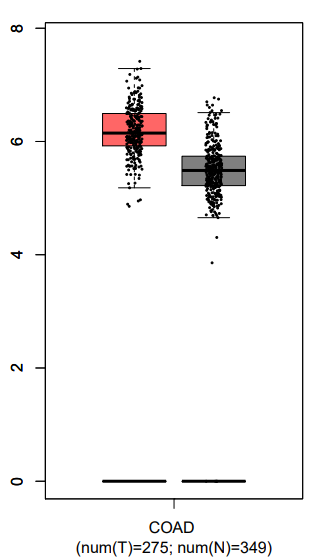


**FAM120A**


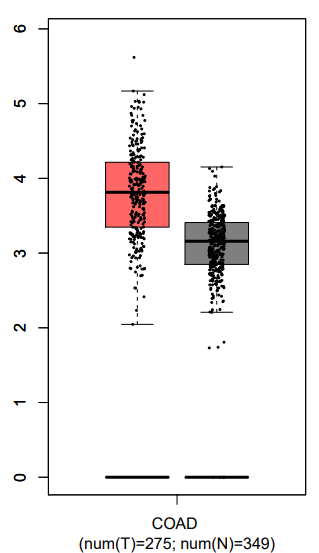


**GEMIN4**


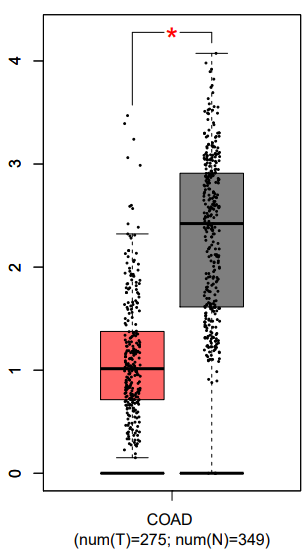


**GNAL**


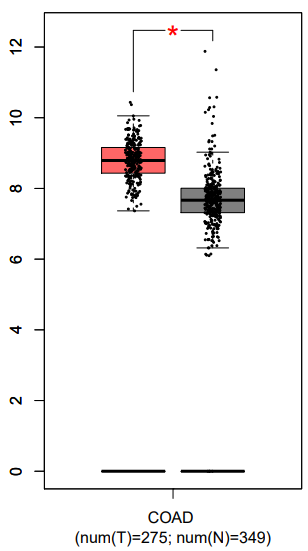


**HSP90A1**


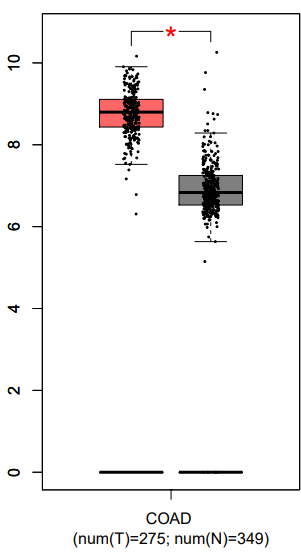


**HSPD1**


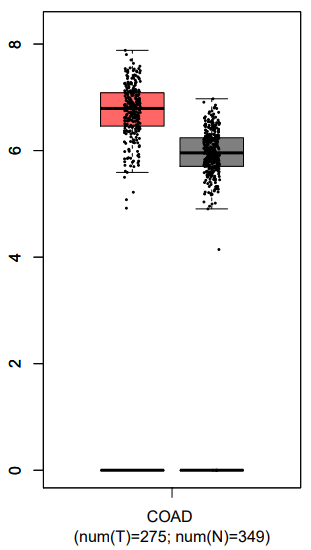


**KPNB1**


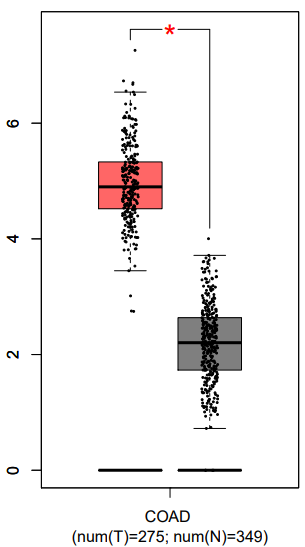


**MET**

**MGAT5**


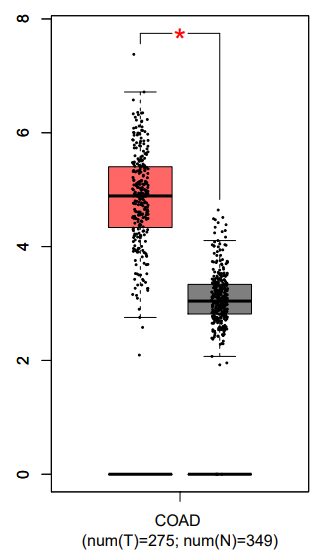

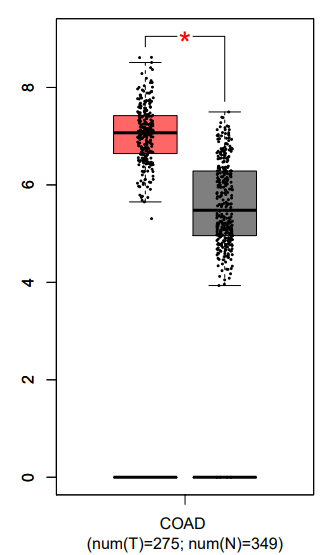


**MLEC**


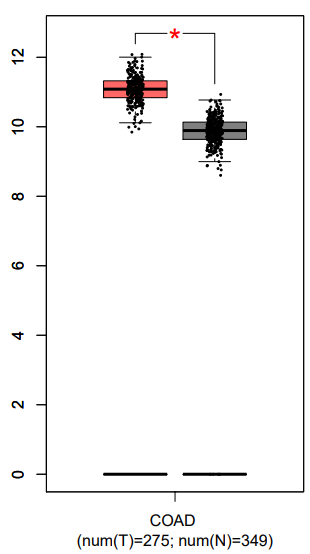


**PTMA**


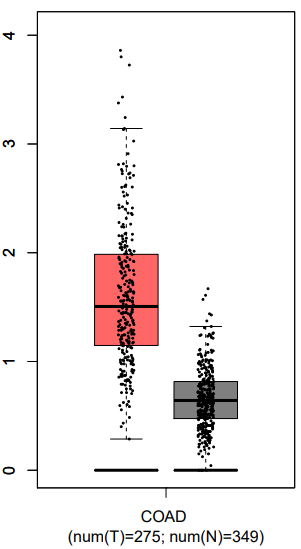


**RNFT2**


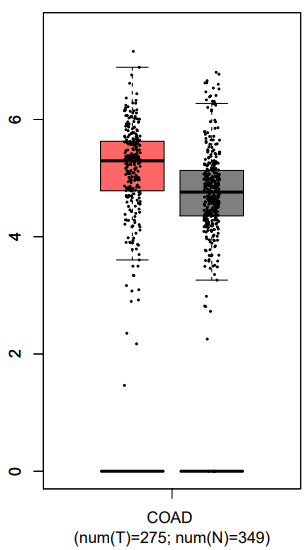


**SLC16A1**


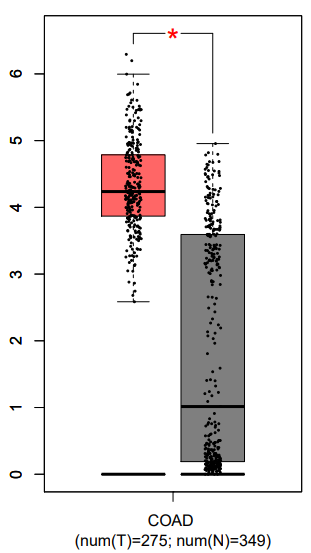


**UGT8**


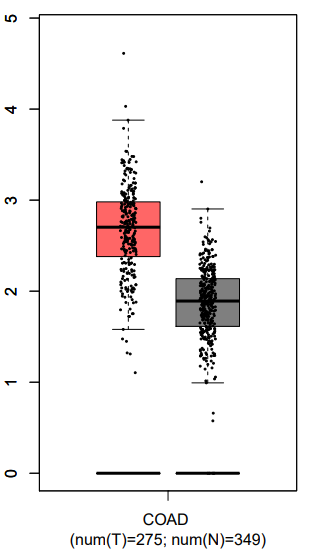


**UTP20**


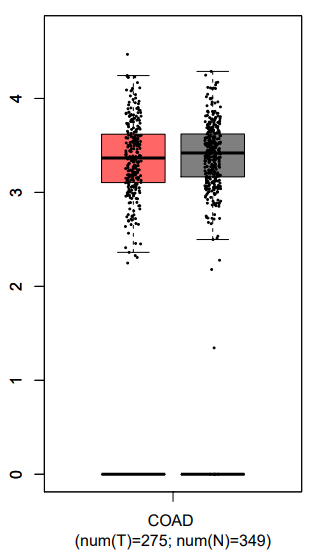


**ZNF33A**


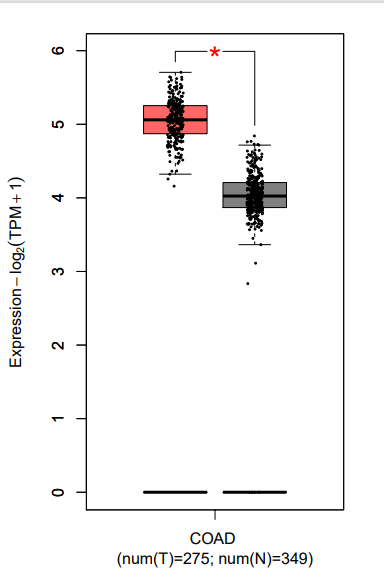


**Supplementary Figure 2.** RNA expression profiles of 17 genes obtained by matching the 177 Wnt target genes (Figure 2G) regulated by IGF2BP1 with iCLIP IGF2BP1 interactome gene list obtained in DLD1-GFP cells. Red bars indicate expression in COAD patient tumor samples while grey bars denote the expression of given genes in normal samples. GEPIA.2 online tool was used for analysis and construction of box plots and 275 COAD tumor samples were compared with 349 normal colon samples.


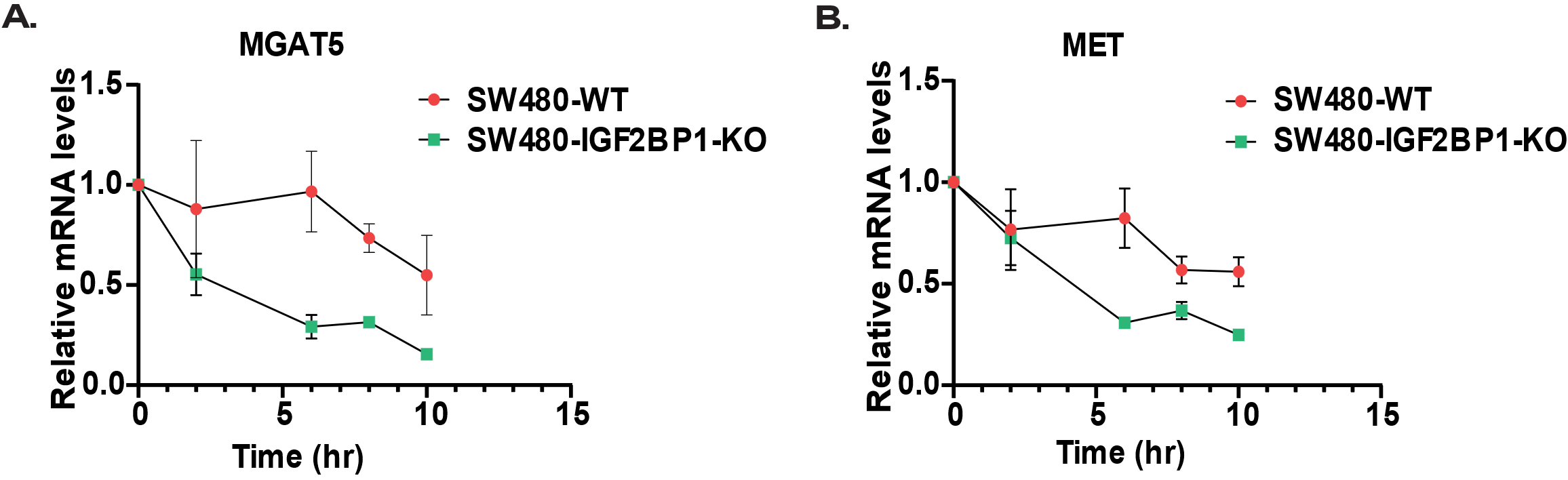


**Supplementary Figure 3.** The mRNA degradation assay in SW480-WT and SW480-IGF2BP1-KO cells. Cells were grown for 2 days and then Actinomycin D was added at time 0, and cell samples were collected at 0-, 2-, 4-, and 8-h time points from the same plate. MGAT5 and MET mRNA levels were evaluated via quantitative RT-PCR. The data are means ± S.D. (*error bars*) of two independent experimental repeats.


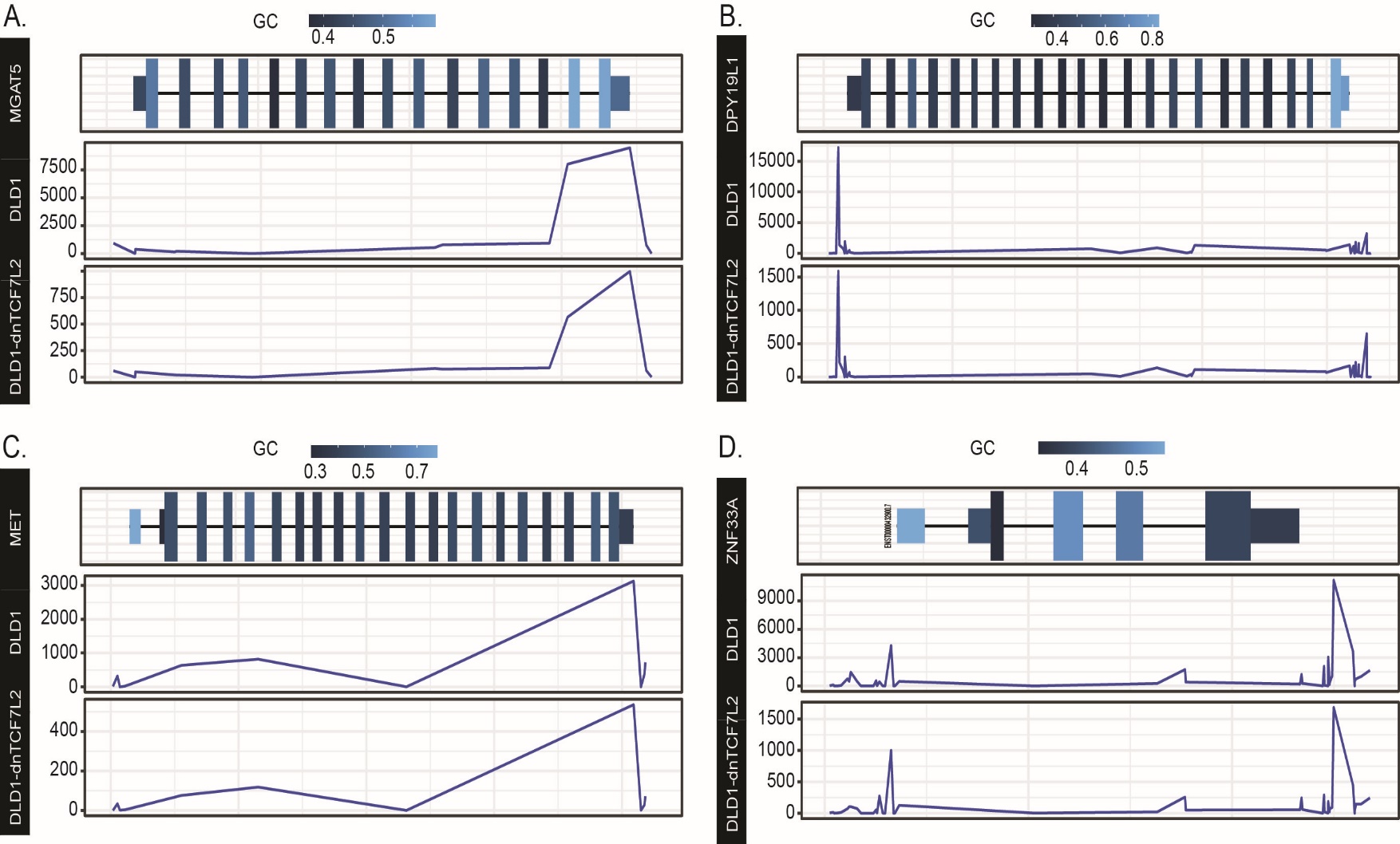


**Supplementary Figure 4.** Output from genCov displays coverage (bottom plots) showing Igf2bp1 binding site on mRNA of MGAT5, DPY19L1, MET, and ZNF33A in samples from DLD1 (Medium plot) and DLD1-dnTCF7L2 (bottom plot). GC content (top plot) is encoded via a range of colors for each exon.


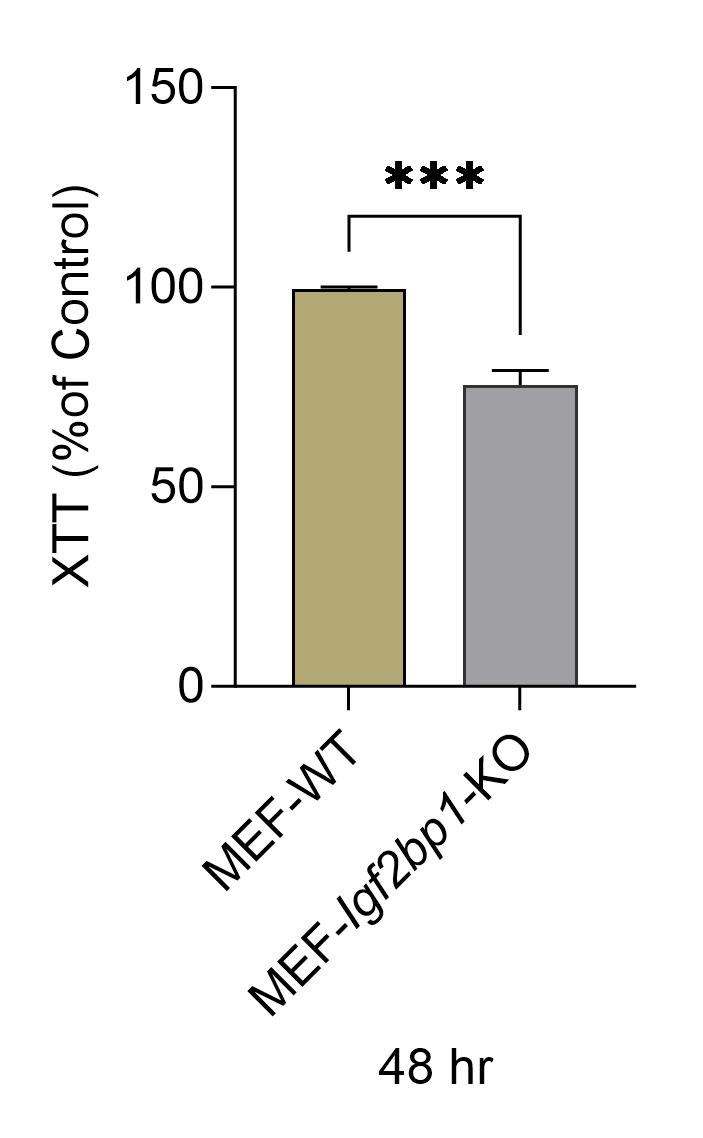

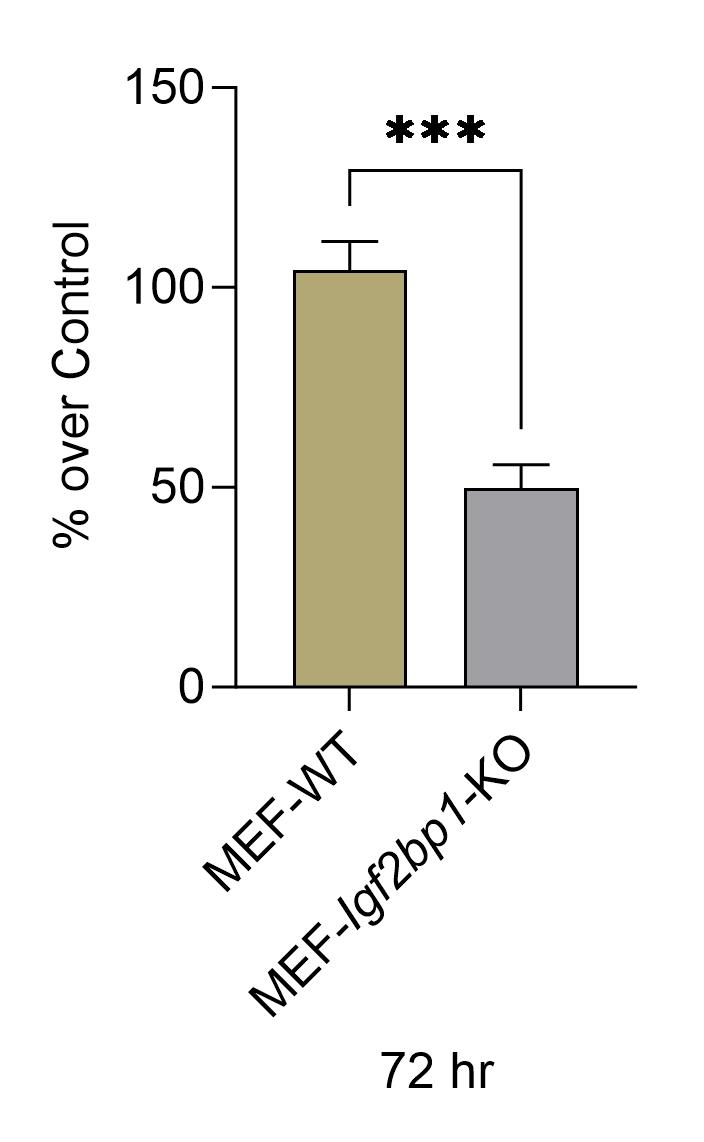


**Supplementary Figure 5.** XTT assay. Cell proliferation after 48 h and 72 h of seeding 5000 MEF-WT and MEF-Igf2bp1-KO cells/well of 96 well plate. Data are means ± SEM. Three experimental replicates per cell type. *: 0.05 > p ≥ 0.01; **: 0.01 > p ≥ 0.001; Student´s t-test.


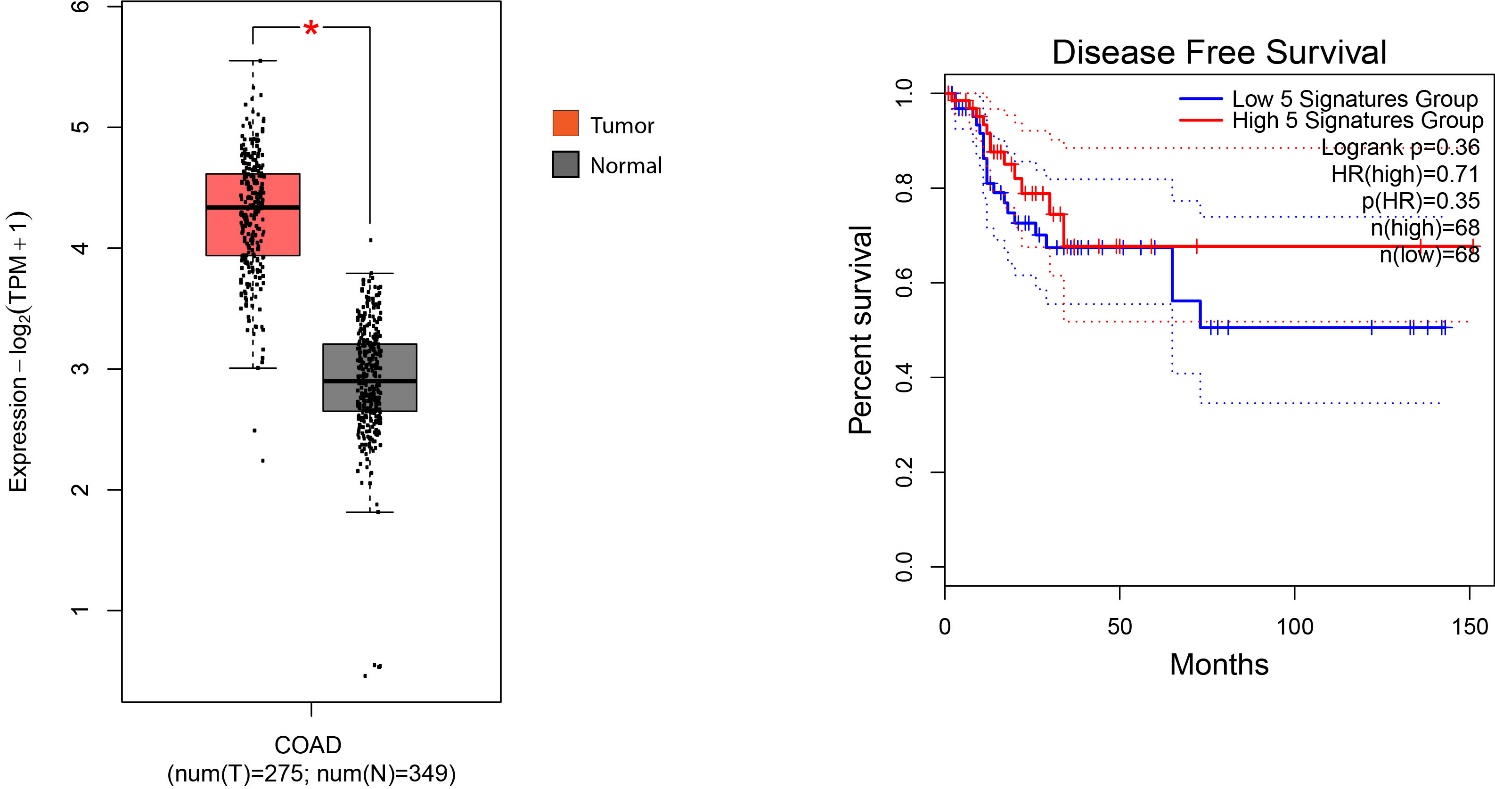


**Supplementary Figure 6.** (A) The 17 random genes from the list of 61 genes that are regulated by Wnt signaling alone (Shown in Fig. 1F) were collectively analyzed using GEPIA2 to assess their RNA expression in tumor versus normal tissue of the COAD dataset. (B) Kaplan-Meier curve plotted to evaluate the disease-free survival of COAD patients expressing the 5-gene signature (picked randomly from the above mentioned 17 genes in Supplementary Figure 6-A), using quartile group cutoff (analysis performed using GEPIA2).
